# Incorporating a neural network into the AMOEBA polarizable force field for ligand field effects of Cu^2+^ ions

**DOI:** 10.1039/d6sc03899a

**Published:** 2026-07-23

**Authors:** Zhecheng He, Yanxing Wang, Shubham Chatterjee, Jean-Philip Piquemal, G. Andrés Cisneros, Jay W. Ponder, Pengyu Ren

**Affiliations:** a Department of Biomedical Engineering, The University of Texas at Austin Austin Texas 78712 USA pren@utexas.edu; b Department of Chemistry and Biochemistry, The University of Texas at Dallas Richardson Texas 75080 USA; c Sorbonne Université, LCT, UMR 7616 CNRS Paris France; d Department of Physics, The University of Texas at Dallas Richardson Texas 75080 USA; e Department of Chemistry, Washington University in St. Louis St. Louis Missouri 63130 USA

## Abstract

Characterizing the solvation of open-shell transition metal ions remains a challenge for classical force fields due to ligand field electronic effects. Here, we present AMOEBA + NN, a machine-learning-augmented polarizable potential, to investigate Cu^2+^ solvation in NH_3_ and H_2_O and mixed environments. The model, trained on quantum-mechanical (QM) association energies, accurately reproduces the Cu^2+^ energy landscape across diverse coordination geometries. Molecular dynamics simulations demonstrate that AMOEBA + NN successfully captures the electronically driven Jahn–Teller (JT) distortion. Evidence from radial distribution functions (RDFs) and geometry optimizations reveals characteristic axial elongation, which is absent in conventional classical descriptions. Kinetic analysis shows a highly dynamic Cu^2+^–NH_3_ environment with a rapid ligand exchange (residence time of 66.52 ps), contrasting with the H_2_O system where binding is two to three orders of magnitude more stable. Furthermore, the calculated hydration free energy of −477.59 ± 0.50 kcal mol^−1^ shows excellent agreement with experimental data (within 1.31 kcal mol^−1^). This work provides a unified, computationally efficient framework for describing the structural, dynamic, and thermodynamic observables of transition metal coordination.

## Abstract 2

Characterizing the solvation of open-shell transition metal ions remains a challenge for classical force fields due to ligand field electronic effects.

## Graphical abstract

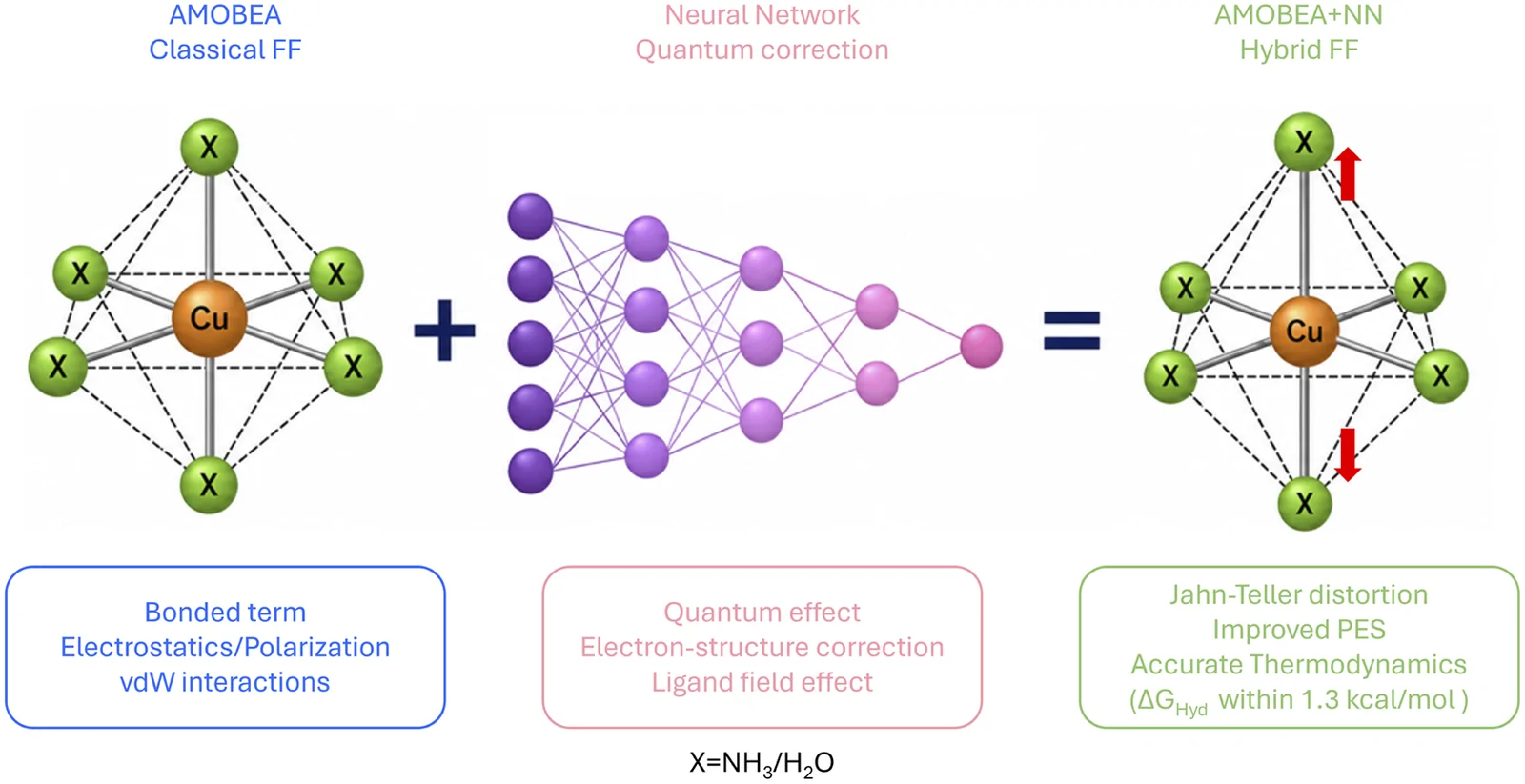

## 1 Introduction

Traditional empirical force fields (FFs) have long served as crucial tools for studying biomolecular and condensed-phase systems.^1–12^ Their use has been proven to be a highly efficient approach to elucidate diverse biological phenomena, such as ligand binding affinity in human serum albumin,^13–15^ conformational dynamics of intrinsically disordered proteins,^16–18^ ion conduction and selectivity mechanisms in potassium channels,^19–21^*etc.* In the biosphere, transition metal ions play a significant role in physiological functions, including electron transfer,^22,23^ stabilizing structures,^24,25^ hydrolysis catalysts,^26,27^*etc.*^28–32^ Copper ions (Cu^2+^) are important and representative transition metal ions that serve as key components in metalloproteins. Their dysregulation is associated with diseases such as neurodegeneration and cancer; therefore, accurate modelling of Cu^2+^ is critical for understanding metalloprotein function and developing metal-based therapeutics.^30,33,34^ However, transition metal complexes pose a fundamental challenge to classical force-field description due to the presence of partially filled d-orbitals, which give rise to strong, highly anisotropic electronic responses that depend sensitively on local coordination geometry. In particular, short-range quantum mechanical effects—such as coordination-number-dependent bonding, ligand field-induced symmetry breaking, and Jahn–Teller (JT) distortions—play a dominant role in determining equilibrium structures and energetics. These effects cannot be adequately represented by isotropic Lennard-Jones potentials, fixed-point electrostatic interactions, or harmonic bonding terms. As a result, key features of transition metal chemistry remain poorly described. Several methods have been proposed to treat transition metal ions within classical molecular mechanics: (1) non-bonded soft-sphere models,^35–39^ (2) the covalent bond approach,^40,41^ (3) dummy-atom models^42,43^ and (4) ligand field molecular mechanics (LFMMs).^44–48^ The non-bonded model is the simplest approach, describing metal–ligand interactions solely through electrostatic and van der Waals (vdW) terms with predefined functional forms. Due to the intrinsic isotropy of non-bonded interaction terms, this model is fundamentally limited in representing the complex multinuclear metal centers. Consequently, it often yields unsatisfactory predictions for metal–ligand binding free energies and metal–ligand equilibrium distances.^49,50^ Polarizable force fields such as AMOEBA^37–39,51^ or the polarizable Gaussian multipole model^52,53^ substantially improve the treatment of electrostatics and many-body polarization. However, their functional forms remain inherently classical and lack an explicit mechanism to encode the local, coordination-specific electronic effects that govern transition metal bonding in the short range. In the covalent model, metal–ligand bond parameters are predefined, allowing an accurate description of known metal binding sites when carefully optimized parameters are available.^54^ However, because these bonds are explicitly fixed, they cannot describe bond breaking or formation, which precludes the identification of previously unknown binding sites, rendering it highly system-specific and poorly transferable.^40,55^ Dummy atom models introduce metal coordination by distributing interaction sites around the metal center in a predefined spatial arrangement. While this strategy can stabilize a target coordination geometry, it relies on artificially introduced sites and nonphysical geometric constraints that are not explicitly derived from the underlying electronic structure. The model cannot adapt to changes in the coordination number, ligand identity, or the local environment either.^56^ The LFMM extends classical force fields by incorporating the ligand field effect term to describe transition metal coordination; however, its reliance on system-specific parameterization and predefined functional forms limits its transferability and applicability to complex, dynamic environments.^57^

Recently, neural network potentials (NNPs) have emerged as a promising alternative to traditional force fields.^58–73^ NNPs are able to learn the potential energy surface (PES) from QM reference data and at the same time maintain transferability and scalability from locality assumption.^59^ NNPs represent the total energy as a sum of atomic contributions, where each atomic energy depends on a local descriptor that captures the chemical environment surrounding the atom with a specified cutoff. Because of this local formulation, NNPs are unable to effectively describe long-range interactions, which restricts their direct application to large-scale molecular dynamics simulations. A widely adopted solution is to combine NNPs with classical force fields,^74–76^ using the NNP to target short-range interactions within a cutoff distance and treating all other interactions *via* the classical force field.

Wang *et al.*^77^ incorporated the ANI-like NNP framework neural network into the AMOEBA force field, known as the AMOEBA + NN framework. In this integrated hybrid framework, short- and long-range noncovalent interactions are treated using the AMOEBA potential, whereas the neural network potential is responsible for modelling the remaining local covalent effects. While this strategy has been validated for organic molecules, its extension to transition metal complexes, where electronic structural effects are critical, remains unexplored.

In this work, we present a neural-network-augmented AMOEBA model specifically designed for Cu^2+^ complexes with ammonia and water ligands. A single-atom neural network acting exclusively on the Cu center, with atom environment vectors (AEVs) constructed only for copper, provides an energy correction for AMOEBA. By restricting the neural network to the Cu^2+^ ion, the model explicitly targets short-range quantum mechanical contributions—such as ligand field effects and Jahn–Teller distortions—while preserving the physically grounded long-range electrostatics and polarization described by AMOEBA. By training on high-level quantum mechanics association energies and validating against structural and dynamic properties, we demonstrate that this physically informed hybrid model accurately reproduces Jahn–Teller distortions, coordination energetics, and finite-temperature behaviour across Cu–NH_3_, Cu–H_2_O, and mixed-ligand complexes.

## 2 Methods

### 2.1 Neural network design

The total energy functional form of AMOEBA + NN is defined as follows:

1 *E* = *E*^NN^ + *E*^AMOEBA^ = *E*^NN^ + *E*_vdW_ + *E*^perm^_ele_ + *E*^ind^_ele_ + *E*_bond_

The rationale for this hybrid framework is to utilize the strengths of both components. In this scheme, AMOEBA provides a physically motivated description of non-bonded interactions across different length scales, including van der Waals forces, permanent electrostatics represented by atomic multipoles, and many-body polarization. And the neural network term is designed to account for deficiencies from QM energies that are not fully captured by AMOEBA.

In this approach, each atom *i* of element X is represented by an atomic environment vector (AEV), *G*^X^_*i*_, which captures local radial and angular chemical features. These descriptors serve as inputs to element-specific multilayer perceptrons, following the general paradigm introduced by Behler and Parrinello and later refined in the ANI framework.^58,60,78–80^ The ANI series, through systematic improvements in descriptor design and training strategies, has achieved near CCSD(T)-level accuracy across diverse chemical properties. Building on this established architecture, we employ the same representation for the neural network component.2
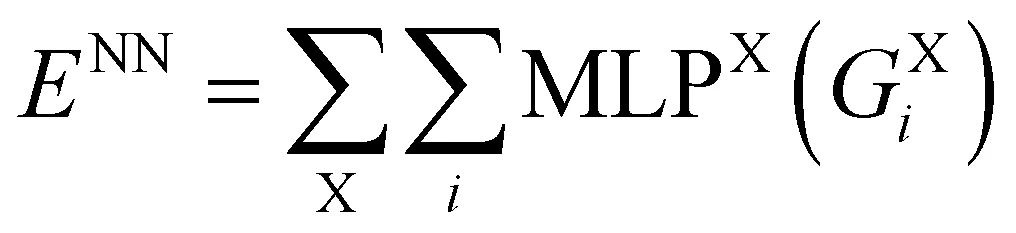
where *i* is the index over all atoms of element X ∈{X} and {X} represents the set of elements covered by the model. The MLPs have three hidden layers and one output layer of 128, 64, 32, and 1 node(s), respectively. All covered elements share the same architecture, but each has its own parameters that are trained independently. The overall mathematical form for one of the MLPs is given by

3 MLP^X^(*G*^X^_*i*_) = *W*^X^_4_(*c*(*W*^X^_3_*c*(*W*^X^_2_*c*(*W*^X^_1_*G*^X^_*i*_ + *b*^X^_1_) + *b*^X^_2_) + *b*^X^_3_)) + *b*^X^_4_

where *c* is the CELU^81^ activation function to add nonlinearity into MLPs. The mathematical definition of CELU is defined as4
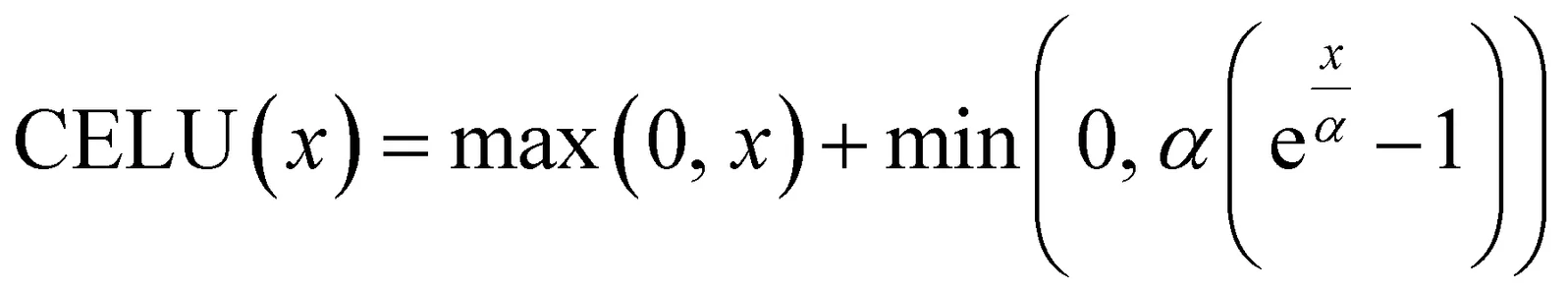
where *α* is used as 1 in this model. *G*^X^_*i*_ is the AEV of the atom *i*. *W*^X^ and *b*^X^ are the trainable weights and biases of the network. To ensure physical consistency, we adopt a bias-free MLP architecture, enforcing the strict constraint that the correction must vanish when the input features are zero, *i.e.*, *b*^X^ = 0. In addition, it is noteworthy that we do not adopt the model ensemble approach utilized in later ANI models, *e.g.*, ANI-1x. In this work, the NN energy employs only one neural network for the Cu element instead of an ensemble of eight networks as in ANI-1x, to keep the computational cost low.

### 2.2 Training details

The association energy of a single copper complex is defined as

5 *E*_assoc_ = *E*_Cu(L)_*n*__ − *E*_Cu^2+^_ − *n* × *E*_L_

where *E*_Cu(L)_*n*__, *E*_Cu^2+^_, and *E*_L_ are the total electronic energies of the Cu(L)_*n*_ complex, Cu^2+^ ion and single relaxed ligand (NH_3_ or H_2_O). Thus, the training target energy of the neural network is defined as6
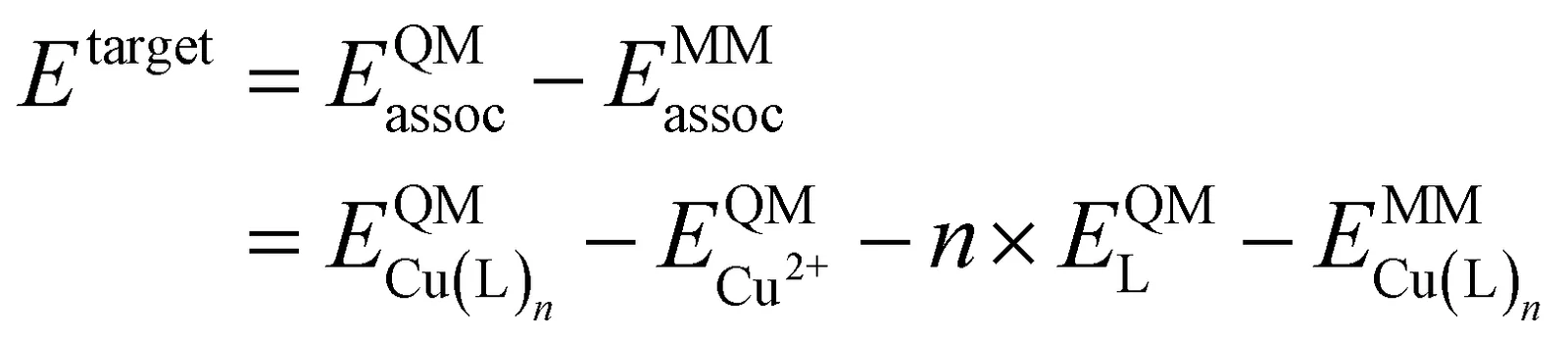


The AMOEBA association energy was defined using the same fragment reference as the QM association energy, but the isolated 
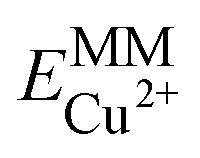
 and *E*^MM^_L_ are taken as zero since the ligand is geometry optimized. The NN target is, therefore, the residual between the QM and AMOEBA association energies for the same cluster geometry. This formulation of relative association energy removes the absolute energy offsets with isolated species, explicitly includes ligand–ligand many-body interactions, and captures the coordination-number and composition-dependent stabilization effects. As the combined AMOEBA + NN potential is ultimately intended for molecular dynamics simulations, atomic forces were also explicitly included as training targets for the neural network component:

7 *F*^target^_*i*_ = *F*^QM^_*i*_ − *F*^MM^_*i*_

where *i* denotes the atom index. The loss function of a given geometry is defined as the sum of the energy loss function and scaled force loss function:

8 *L* = *L*_E_ + *αL*_F_

9 *L*_E_ = (*E*^NN^ − *E*^target^)^2^

10
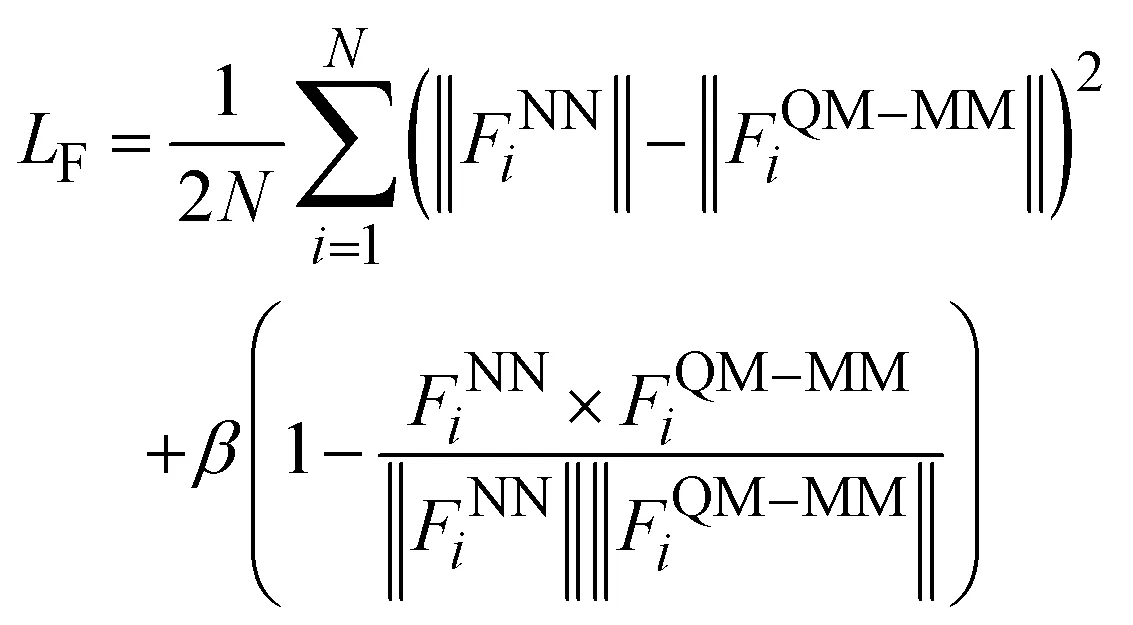
where *α* is a hyperparameter to balance between the force and energy losses, loss of energy *L*_E_ is the square of NN energy and reference energy difference, and loss of force *L*_F_ is defined as the average over all atoms of the weighted mean of the cosine distance and the squared magnitude error between the predicted and QM reference atomic force vectors. The hyperparameters in the loss function were set to *α* = 0.1 Å^2^ and *β* = 100 kcal^2^ mol^−2^ Å^−2^. *α* was adopted from the ANI model, and *β* was determined empirically from previous study.^77^ The Adam^82^ optimizer implemented in PyTorch is used with the learning rate scheduled to decay by 20% from the starting value of 0.005 after plateauing for 100 epochs. The training is stopped early if the loss has plateaued for 50 epochs.

We employ a single-atom neural network acting only on Cu. Atomic environment vectors are constructed exclusively for the copper atom, encoding the geometric arrangement of surrounding ligands within a 3.5 Å cutoff for radial and angular directions. Due to the excessive number of degrees of freedom introduced by hydrogen atoms, only heavy atoms are probed by the network. All quantum data are used for a 5-fold cross validation scheme training. During model training, the 5-fold cross-validation procedure was used to monitor training stability and interpolation performance within the sampled Cu^2+^ coordination dataset. In each fold, 80% of the configurations were used for training and the remaining 20% were used as a validation subset. The folds were constructed to maintain representation of the major ligand compositions, coordination numbers, and sampling sources across the training and validation subsets. This validation protocol was intended to assess model consistency within the sampled coordination manifold and to reduce the risk of overfitting during training; it was not designed as a strict extrapolation test for unseen ligand chemistries or completely independent trajectories. The AMOEBA09 force field is used, with AMOEBA energy and gradients calculated using the Tinker and Tinker-GPU software.^83,84^ Quantum data, trained model and training details are accessible from https://github.com/prenlab/amoeba_nn. Details can be found in ‘AMOEBA + NN: Metal Ion Model’ in the README.md file on GitHub.

### 2.3 Quantum mechanics benchmark and reference level selection

To define a reliable quantum reference for training, we benchmarked several electronic structure methods on randomly selected Cu^2+^ complexes coordinated by four, five, and six ligands. For each coordination environment, five representative structures were selected. Association energies were evaluated at different levels of theory, and the average deviation relative to CCSD(T) reference energies is shown in Fig. 1. The tested methods include: ωB97X-D3, B3LYP D3BJ, and PBE0 D3BJ with the def2-TZVP basis set, Resolution of the Identity-MP2 (RI-MP2) with def2-SVP, def2-TZVP, and def2-QZVP basis sets, and CCSD(T) with def2-QZVP as the reference standard. Based upon the results, MP2/def2-TZVP was selected as the reference level for training data, offering a balanced compromise between accuracy and computational cost. All QM energies and gradients used for training are computed at this level. And all QM calculations are performed using the ORCA-6.1.1 software.^85–90^

**Fig. 1:**
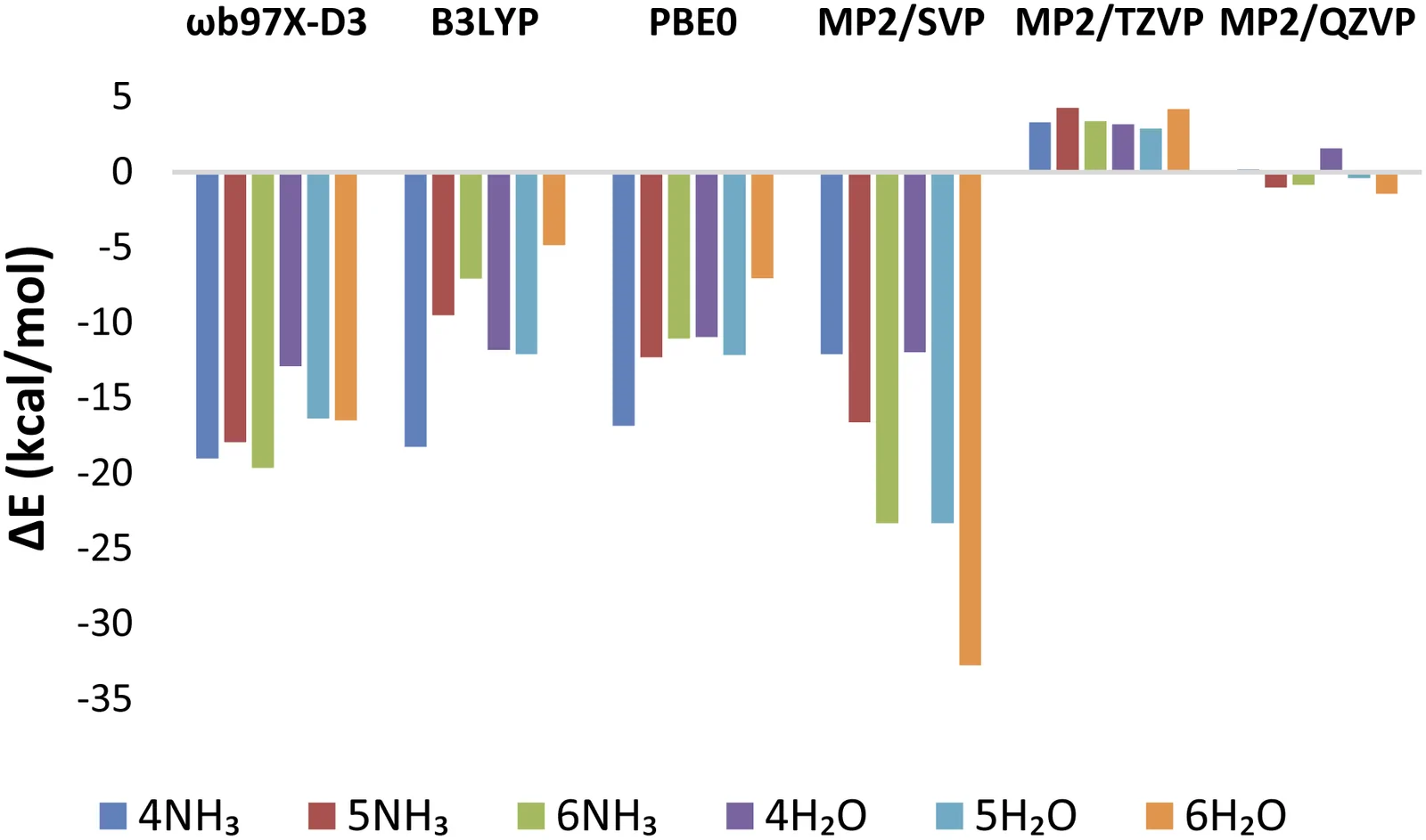
Average energy deviation of different species relative to the CCSD(T) reference.

### 2.4 Data sampling

There are two sampling methods investigated: (1) Monte Carlo-like sampling for 4, 5, and 6 coordinated environments with pure NH_3_ and pure H_2_O: we started from MP2-optimized Cu^2+^ complexes, and the copper coordinate is fixed and each Cartesian coordinate of each ligand heavy atom was independently perturbed by a random displacement sampled from a uniform distribution between −0.5 and 0.5 Å to ensure physically relevant distortion and exclude unphysical high-energy structures. Structures with single point energies exceeding 30 kcal mol^−1^ above the minimum are discarded. This protocol yielded over 50 000 configurations. To reduce computational cost, all energy calculations in this method are conducted at the ωB97X/def2-SVP level of theory using PySCF with GPU acceleration^91–94^ and (2) *Ab initio* molecular dynamics (AIMD) simulations at 200 K/240 K/300 K performed with three different levels of theory combinations: MP2/def2-SVP using ORCA-6.1.1 and B3LYP/6-31G(d)_LANL2DZ and HF/6-31G(d)_LANL2DZ as computed using TeraChem,^95–98^ including Cu(NH_3_)_*n*_ (*n* = 1, 2, 3, 4, 5, 6) with approximately 200 000 samples at the MP2/def2-SVP level of theory, Cu(H_2_O)_*n*_ (*n* = 1, 2, 3, 4, 5, 6) with approximately 140 000 samples at the B3LYP/6-31G(d)_LANL2DZ level of theory, and Cu(NH_3_)_*n*_(H_2_O)_6−*n*_ with approximately 50 000 samples at the HF/6-31G(d)_LANL2DZ level of theory.

In addition, to sample the energy pathway more thoroughly from high-energy configurations to local minima, geometry optimizations were performed on approximately 6000 selected structures. Each optimization was carried out for 10–20 steps, yielding additional ∼80 000 configurations. All geometry optimizations were conducted at the HF/6-31G(d) level of theory using the PySCF package.

To better characterize the training data, we analyzed the dataset based on composition, sampling source, Cu-centered AEV distribution, Cu–ligand distances, and ligand–Cu–ligand angles (Table S2 and Fig. S1–S3). The training structures span distinct coordination numbers and ligand identities, and the different sampling protocols provide complementary coverage. We do not assume that every configuration is independently necessary; rather, the large dataset was used to ensure broad coverage of the local Cu^2+^ coordination environments relevant to geometry optimization and condensed-phase simulations.

### 2.5 Hydration free energy

The hydration free energy of Cu^2+^ was computed using an alchemical free energy perturbation (FEP) approach. A total of 21*λ* windows were employed to decouple the solute from the solvent environment. In the first window, the NN contribution was removed to ensure a consistent reference state. Electrostatic interactions were then gradually turned off over the next 10 windows, followed by van der Waals interactions over the remaining 10 windows. We note that removing an NN term in a single step is not guaranteed to be generally valid, because sufficient phase-space overlap between the NN-on and NN-off states must be maintained. In the present hydration calculation, this approximation is relatively well behaved because the NN term acts as a localized Cu centered correction, while the underlying AMOEBA potential already preserves the primary Cu–O coordination shell. Therefore, the NN removal step does not induce a qualitative change in the hydration structure but rather removes a short-range correction from an already stable AMOEBA reference state. For systems in which the NN term produces larger structural rearrangements, multiple intermediate NN coupling windows and explicit overlap analysis would be required.

Before running MD, the initial system was equilibrated for 1 ns at 298 K, 1 bar in the *NPT* ensemble. For each *λ* window, a 5 ns production simulation was performed at 298 K and 1 bar with a 2 fs timestep and coordinates were saved every 2 ps for analysis. Real-space distance cutoff for Ewald summation is set to 7 Å; van der Waals potential cutoff is set to 9 Å. Free energy differences between adjacent windows were calculated using the Bennett acceptance ratio (BAR) method, and the total hydration free energy was obtained by summing over all windows. Standard state corrections were applied following the convention for single-ion solvation free energies, accounting for the proton reference state as described by Schmid^99^ and Jing.^39^ HFE calculations and BAR analysis are conducted using Tinker-GPU.

### 2.6 Molecular dynamics simulations

All AMOEBA molecular dynamics simulations were conducted *via* Tinker-GPU with an NN implementation.^100^*Ab initio* molecular dynamics simulations of Cu^2+^ with bulk NH_3_ were conducted at the ωB97X-D3/6-31G(d)_LANL2DZ level of theory using TeraChem. Radial distribution function data were generated using Tinker v25.

## 3 Results and discussion

### 3.1 Energy prediction

The energy predictive performance of the proposed AMOEBA + NN model was first evaluated in terms of its ability to reproduce quantum-mechanical association energies for Cu^2+^ complexes with ligands. All available configurations generated from Monte Carlo-like sampling, *ab initio* molecular dynamics, and short geometry relaxation trajectories were used collectively as the training data set. 5-fold cross-validation was carried out by partitioning the data set into mutually exclusive subsets, ensuring that structurally similar configurations were distributed across different folds and preventing the validation set from overestimating the model's transferability.

The trained model is used to re-predict association energies for all configurations in the training set. Fig. 2 compares the AMOEBA + NN energies with the corresponding MP2 energies. The correlation is well characterized by a high coefficient of determination (*R*^2^) and a low root mean squared error (RMSE), indicating that the model accurately captures the underlying energy landscape across a wide range of coordination geometries, ligand compositions, and distortion amplitudes. Since the NN contribution is an energy correction rather than an independent force-output model, the AMOEBA + NN forces were obtained analytically as the negative gradients of the total AMOEBA + NN energy. The force consistency was evaluated by comparing the *x*, *y*, and *z* components of the resulting forces on each non-hydrogen atom with the corresponding MP2 reference forces (Fig. S4).

**Fig. 2:**
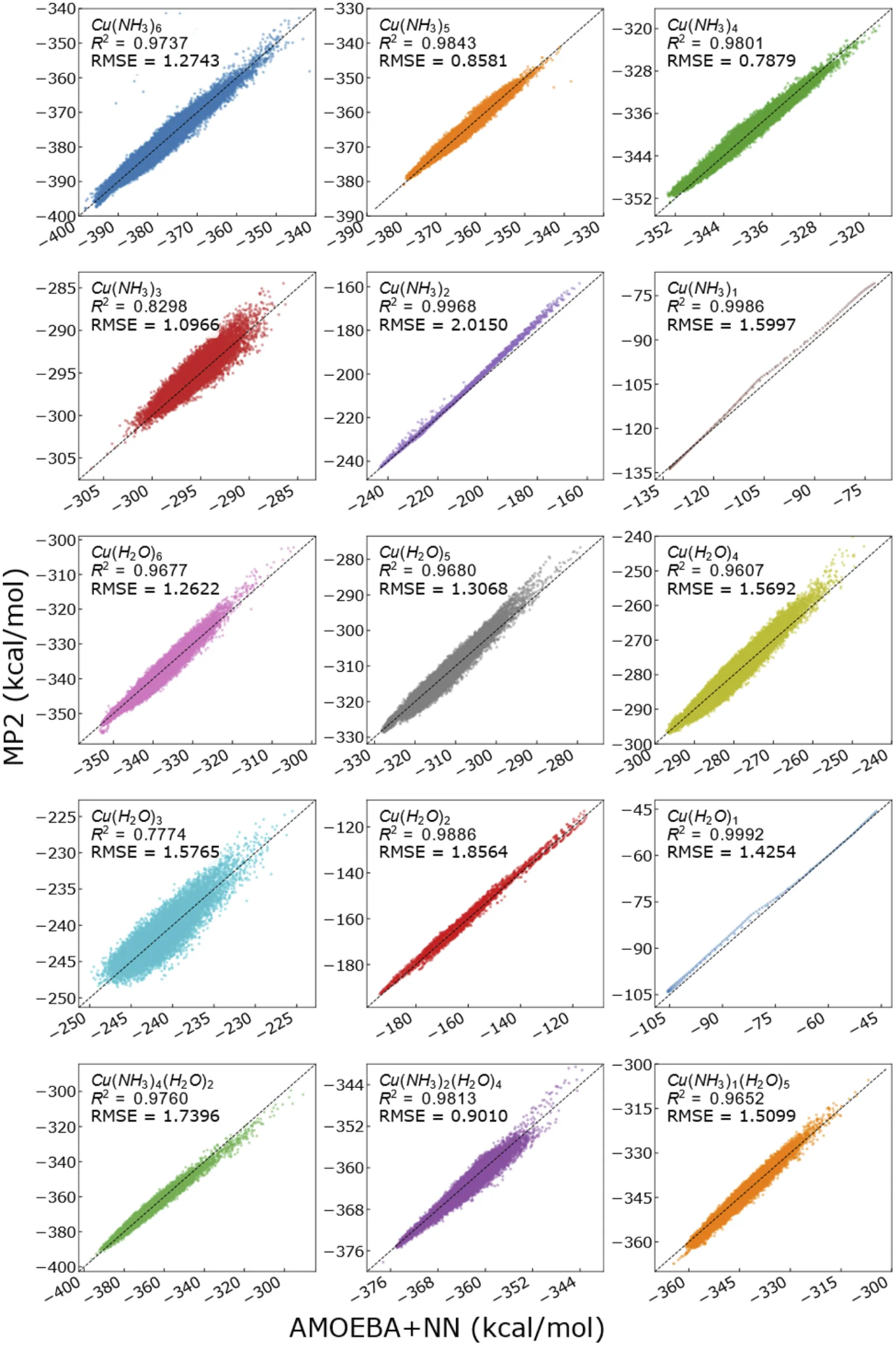
Scatter plots of neural network prediction *vs.* MP2 reference energy. All energies are in kcal mol^−1^. The dotted line denotes the identity line (*y* = *x*).

### 3.2 Geometry optimization

Besides energetic accuracy, geometry optimizations were also performed using the AMOEBA + NN potential for a diverse set of coordination environments to test its capability to reproduce equilibrium geometries. The optimized structures include Cu^2+^ complexes coordinated by three, four, five, and six ligands, as well as mixed-ligand octahedral complexes with a fixed coordination number of six but varying NH_3_/H_2_O composition. Specifically, the mixed-ligand set comprises two Cu(NH_3_)_2_(H_2_O)_4_ configurations, two Cu(NH_3_)_4_(H_2_O)_2_ configurations, and one Cu(NH_3_)(H_2_O)_5_ configuration. Cu(NH_3_)_3_(H_2_O)_3_ and Cu(NH_3_)_5_(H_2_O) complexes fail to retain stable geometry in *ab initio* MD simulations and are therefore not included.

Fig. 3 presents optimized geometries for these complexes. Differences in Cu–ligand bond lengths are observed. In six-coordinate complexes with pure NH_3_ and H_2_O, the model consistently reproduces axial elongation relative to equatorial bonds, a characteristic of the Jahn–Teller effect reflected by the model. Moreover, the magnitude and direction of bond-length asymmetry vary with ligand composition, indicating that the neural network correction captures subtle differences between Cu–NH_3_ and Cu–H_2_O interactions. In mixed complexes, the AMOEBA + NN model reproduces the qualitative axial/equatorial asymmetry, but the magnitude of the Jahn–Teller elongation can be slightly underestimated, as observed for Cu(NH_3_)_4_(H_2_O)_2_. This case is more challenging than pure-ligand complexes because the preferred axial ligands depend on a subtle balance between Cu–N and Cu–O interactions, ligand arrangement, and polarization. The mixed Cu(NH_3_)_4_(H_2_O)_2_ subset is also less represented than the pure six-coordinate datasets, which may reduce the sampling density along the relevant distortion coordinate. Targeted mixed-ligand sampling may further improve the quantitative JT amplitude. Some differences in the orientations of coordinated water molecules are observed between the MP2 and AMOEBA + NN optimized structures. These differences should be interpreted with the scope of the present model in mind. The NN correction is centered on Cu and is designed to correct the local metal and heavy atom donor coordination environment, including Cu–ligand distances, ligand field effects, and Jahn–Teller distortions. In contrast, the rotational orientation of water hydrogens is governed primarily by the underlying AMOEBA bonded terms, permanent electrostatics, and polarization and is not explicitly targeted by the NN descriptor. Therefore, the geometry comparison in Fig. 3 is intended mainly to assess the Cu–ligand coordination geometry rather than to provide a complete benchmark of water orientational degrees of freedom. This limitation may be improved in future models by including a hydrogen sensitive descriptor. These results highlight a key advantage of the present single-atom neural network design: by encoding the local coordination environment around the metal center while retaining a physically grounded energy contributed by AMOEBA, the model reproduces electronically driven geometric anisotropy in the hybrid framework.

**Fig. 3:**
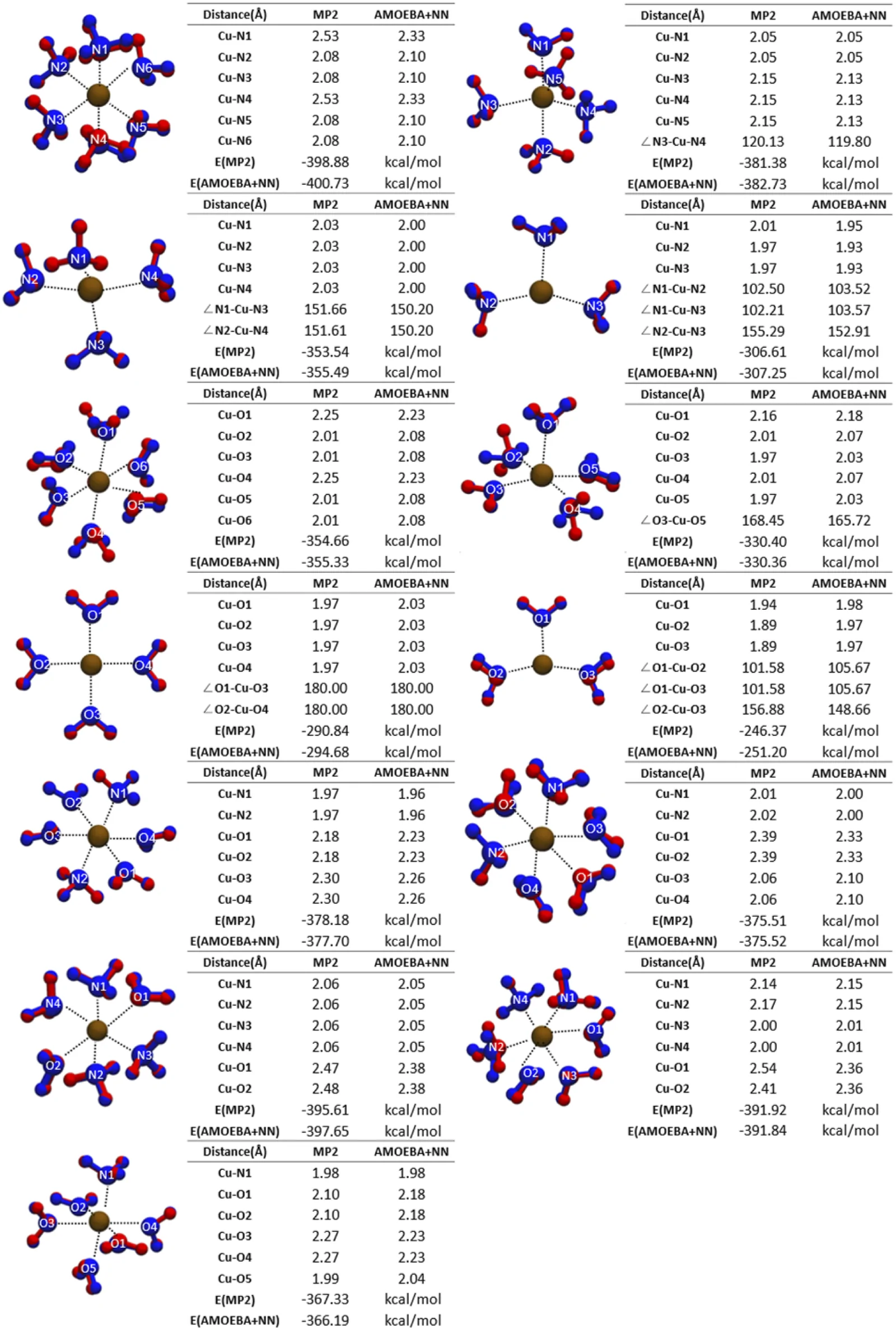
Optimized geometry comparison of QM (blue) and AMOEBA + NN (red) results.

### 3.3 Molecular dynamics simulation

Molecular dynamics simulations were performed to characterize the solvation behavior of Cu^2+^ in pure NH_3_, pure H_2_O, and mixed NH_3_/H_2_O environments using the AMOEBA + NN potential (Fig. 4). Each system was equilibrated under *NVT* conditions for 200 ps in a 30 Å cubic box, followed by 10 ns of *NPT* production simulations at 1 bar. The NH_3_ system was simulated at 200 K, close to the freezing point of liquid ammonia (194.95 K), the H_2_O system was simulated at 298 K and mixed systems were simulated at 240 K.

**Fig. 4:**
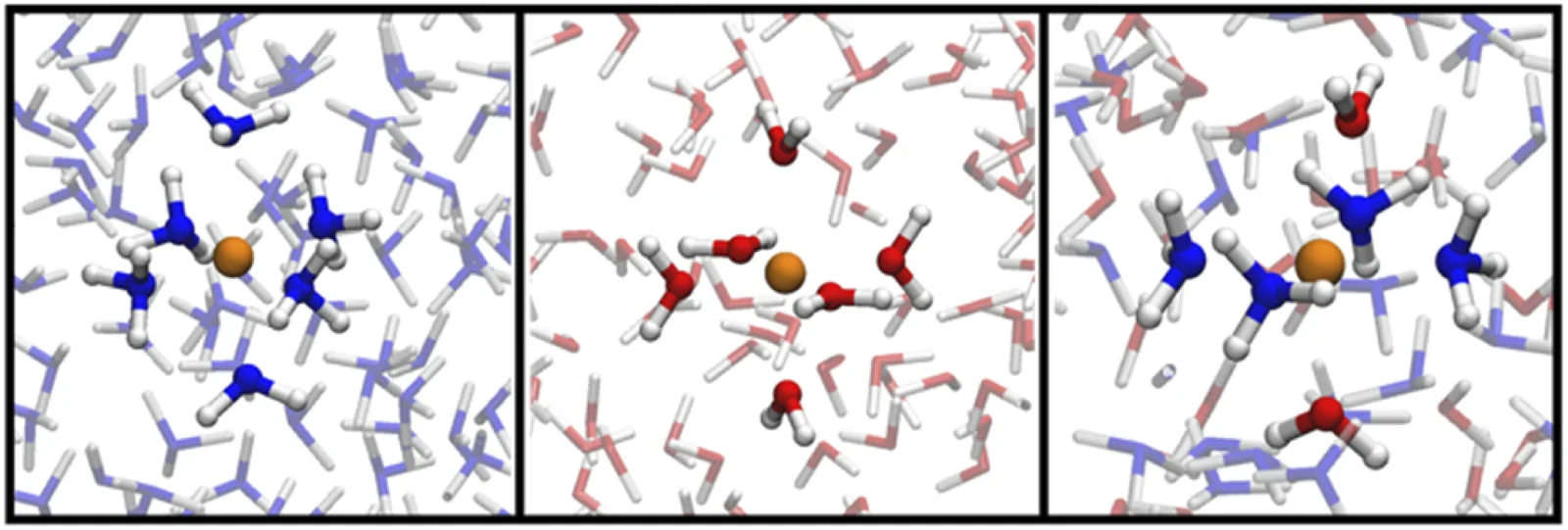
Cu^2+^ in different solvent environments.

The X–Cu–X (X = N, O) angular distributions were analyzed. As shown in Fig. 5, the angles are predominantly centered at around 90° and 170° in both systems, consistent with an overall octahedral coordination geometry with JT distortion. The radial distribution functions (RDFs) and coordination numbers (CNs) reveal a consistent six-coordinate solvation structure across all systems (Fig. 6). In the NH_3_ system, the Cu–N RDF exhibits a clear peak splitting at approximately 2.30 and 2.35 Å, indicating the axial and equatorial bond distribution of the Jahn–Teller (JT) effect. In contrast, classical AMOEBA produces a single symmetric peak at 2.01 Å, failing to capture this structural asymmetry. For the H_2_O system, although distinct peak splitting is not observed, the Cu–O RDF shows noticeable asymmetry and broadening relative to classical AMOEBA, suggesting deviations from ideal octahedral symmetry. In the mixed solvent system, the CNs of NH_3_ and H_2_O plateau at 3.80 and 2.35 respectively, which is consistent with the relatively stable Cu(NH_3_)_4_(H_2_O)_2_ configurations observed in the training data. These results demonstrate that the AMOEBA + NN model captures subtle structural distortions beyond those accessible to conventional non-polarizable descriptions.

**Fig. 5:**
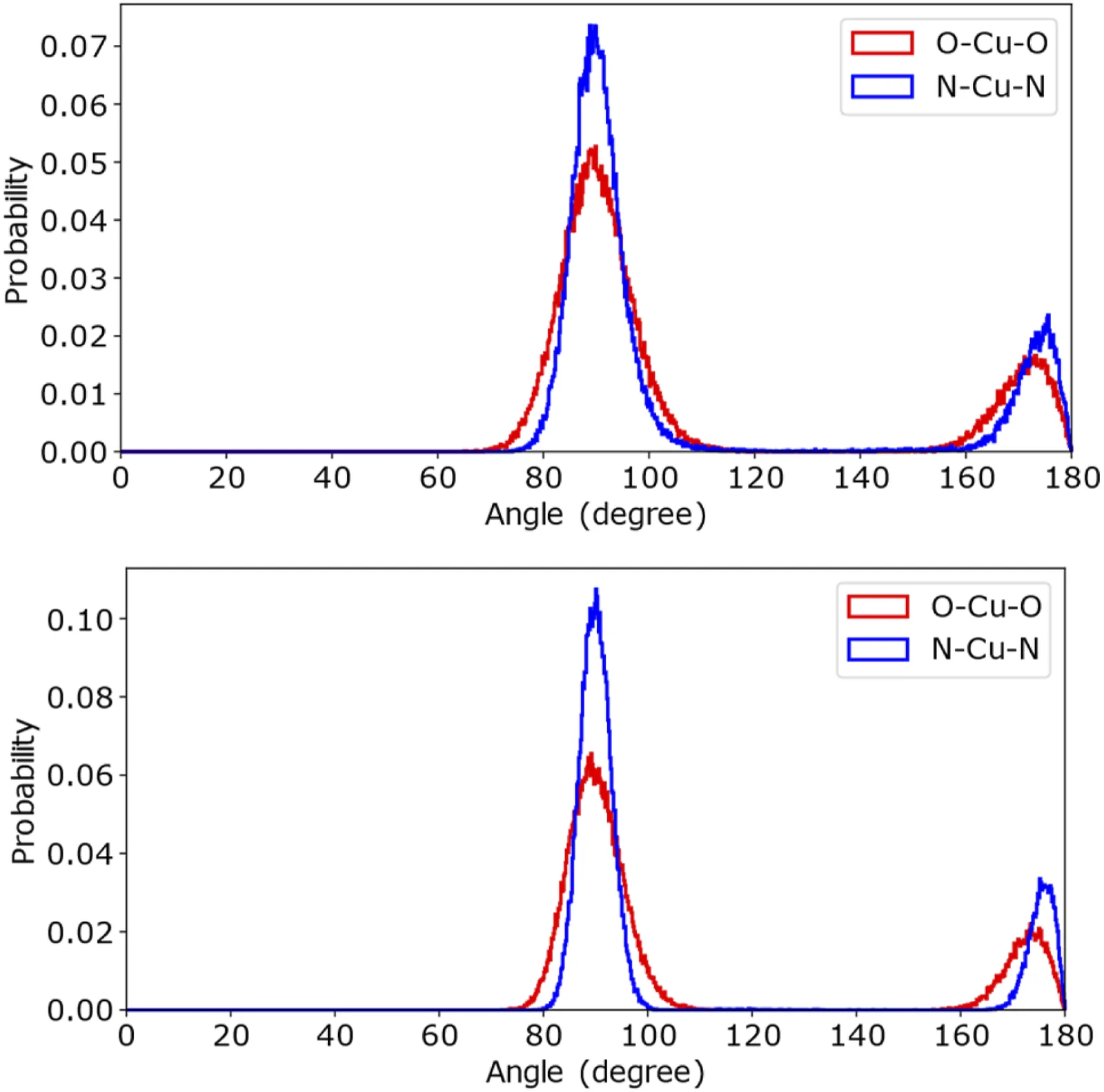
X–Cu–X angle distribution in a pure ligand system during 10 ns of MD simulations (X = N, O) Top: AMOEBA + NN; bottom: AMOEBA.

**Fig. 6:**
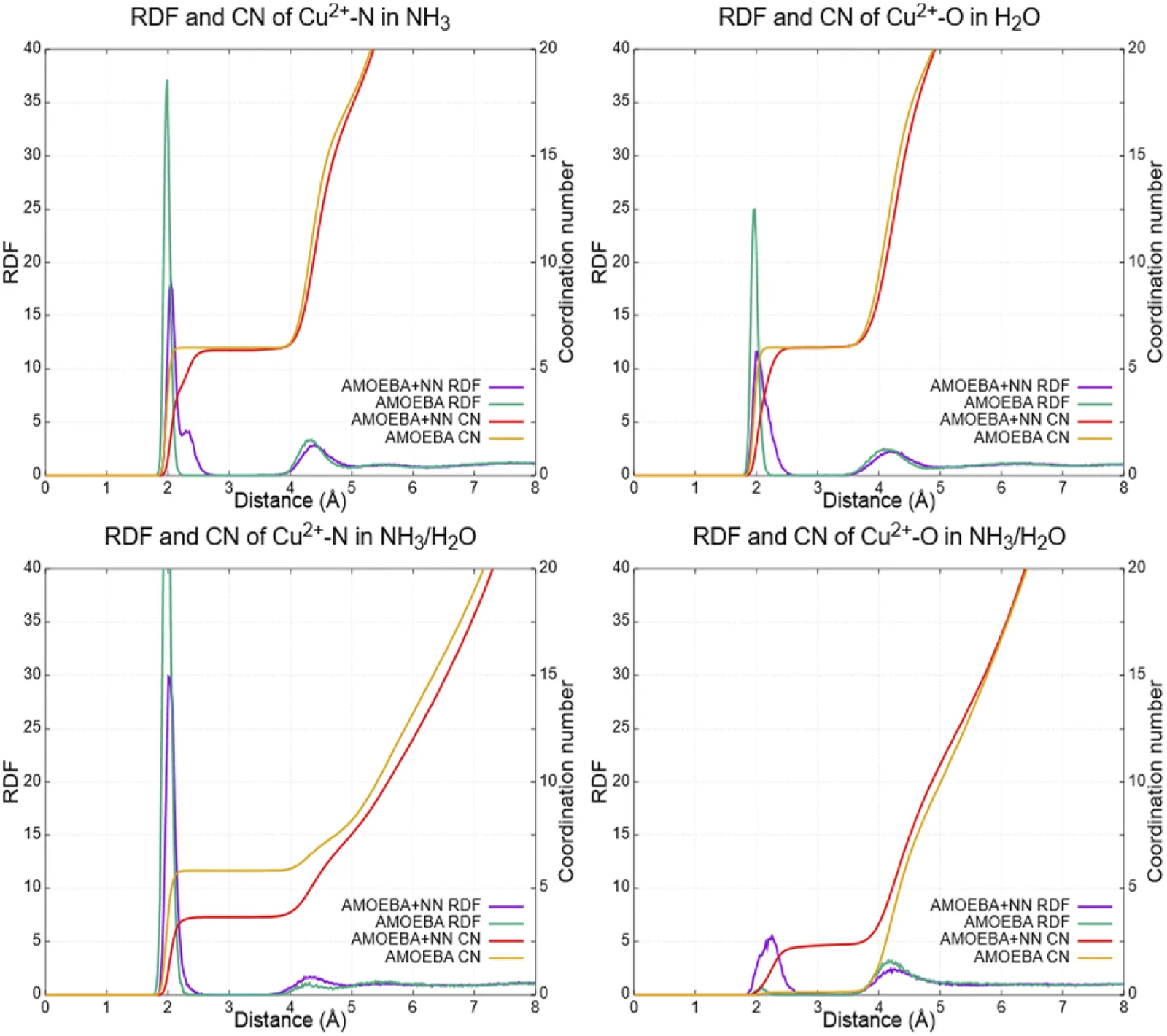
RDF and CN of Cu^2+^ in different solvent systems.

Ligand residence times were further analyzed to quantify solvation stability. By inspecting the starting point of the second solvation shell as indicated in Fig. 6, cutoff distances of 3.6 Å for NH_3_ and 3.0 Å for H_2_O were used. A ‘direct’ method^101^ with 2 ps tolerance time is used to analyze the residence time. For the NH_3_ system, a total of 382 exchange events were identified. The residence time distribution is predominantly characterized by short-lived events, with 72.50% and 17.73% of events occurring at 2.0 ps and 4.0 ps, respectively. Collectively, short-term exchanges (ranging from 2 to 14 ps) account for 97.78% of the total events (Fig. S5). However, the distribution also exhibits a tail due to a small number of persistent coordination states that span the entire simulation timescale (2.22%), which gives an average residence time of 66.52 ps and 2.0 ps as median. This revised value highlights the highly dynamic nature of the NH_3_ coordination environment, characterized by rapid ligand exchange between the first and second solvation shells. In contrast, only 2 ligand exchange events were observed for H_2_O system simulations, 2670 ps and 5480 ps respectively. The residence time of Cu^2+^ is reported from hundreds of picoseconds to few nanoseconds in various studies.^102–105^ Schwenk *et al.*^106^ explored the residence time of Cu^2+^ in liquid ammonia at 10–32 ps by the QM/MM method. A 40 ps *ab initio* molecular dynamics simulation with 30 NH_3_ at 200 K is conducted. Fig. 7 shows the Cu–N distance trajectories from the *ab initio* MD simulation. Ligand exchange events are highlighted by red vertical lines, clearly illustrating the dynamic coordination behavior, with an average residence time of 9.64 ps. Fig. 8 presents the corresponding ligand exchange events from the AMOEBA + NN simulation. For clarity, only ligand exchange events are shown, while the Cu–N distance trajectories are omitted. The NH_3_ system exhibits substantially more frequent ligand exchange than the H_2_O system. These results undoubtedly agree with the conclusion that the residence time of copper in ammonia is by at least two to three orders of magnitude less than that in water, indicating significantly more stable and strong binding ability of H_2_O than NH_3_. To further investigate the differences in binding affinities, rigid scans were performed by pulling a single ligand from the Cu(x)_6_^2+^ complex (Fig. S6). In both systems, axial ligands exhibit weaker binding compared to their equatorial counterparts. Notably, the axial NH_3_ ligand resides in a shallower potential well (∼9 kcal mol^−1^) than H_2_O (∼15 kcal mol^−1^). The energy profiles generated by the AMOEBA + NN model show excellent agreement with the reference MP2 curves, further validating the predictive performance and transferability of our model in capturing subtle ion–ligand interactions.

**Fig. 7:**
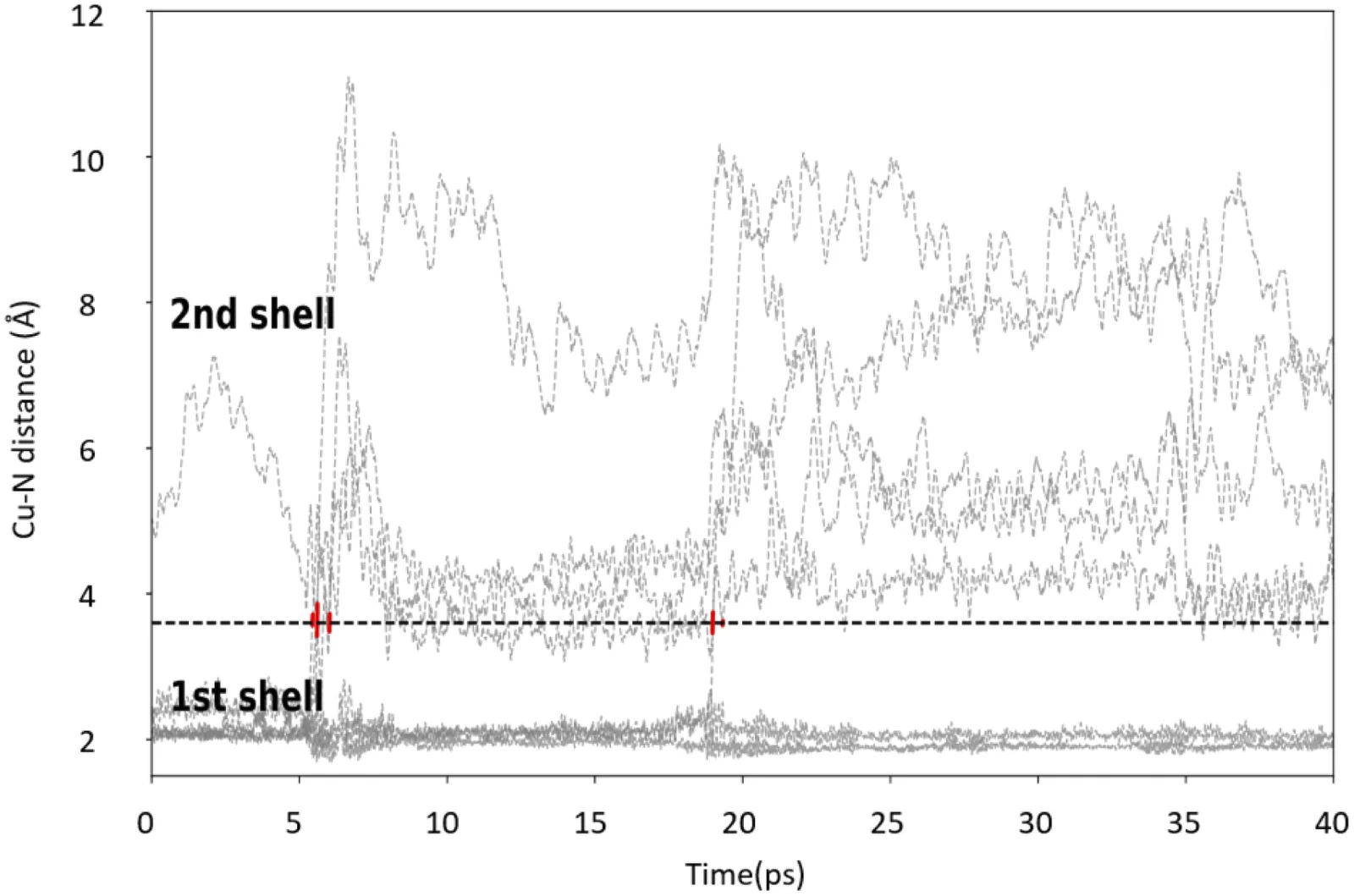
Cu–N distance trajectories from a 40 ps *ab initio* MD simulation of one Cu^2+^ ion solvated by 30 NH_3_ molecules at 200 K. Red marks indicate ligand exchange events, demonstrating the highly dynamic coordination environment of Cu^2+^ in liquid ammonia. The dashed horizontal line denotes the cutoff distance used to identify ligand exchange.

**Fig. 8:**
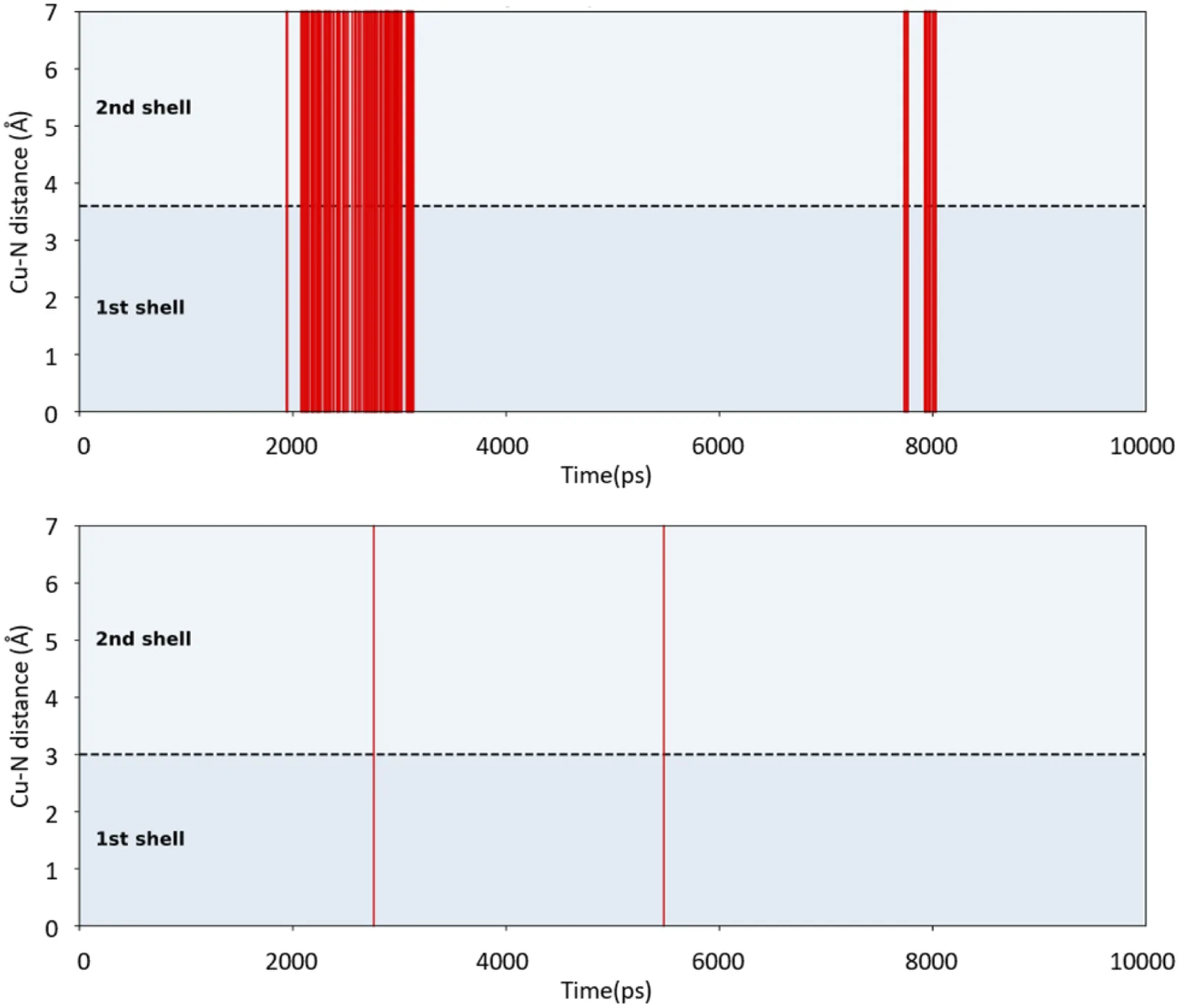
Timeline of ligand exchange events during AMOEBA + NN MD simulations. Each red vertical line marks a ligand exchange event, allowing the exchange frequency to be directly compared between the two systems. The NH_3_ system (top) shows frequent ligand exchange throughout the simulation, whereas only two exchange events are observed in the H_2_O system (bottom), demonstrating the much higher lability of Cu^2+^–NH_3_ coordination. The dashed horizontal line indicates the cutoff distance used to define ligand exchange (3.6 Å for NH_3_ and 3.0 Å for H_2_O).

### 3.4 Hydration free energy

To assess the thermodynamic accuracy of the model, the hydration free energy of Cu^2+^ was computed using free energy perturbation. The classical AMOEBA model yields a hydration free energy of −488.64 ± 0.19 kcal mol^−1^, while inclusion of the NN term introduces a correction of 11.05 ± 0.31 kcal mol^−1^, resulting in −477.59 ± 0.50 kcal mol^−1^. Experimental values reported by Marcus^107^ (−484.70 kcal mol^−1^) were used with an additional correction for the proton reference state energy, as discussed by Jing *et al.*,^39^ leading to a +2.9 kcal mol^−1^ correction term per charge. The final hydration free energy of Cu^2+^ is therefore set at −478.90 kcal mol^−1^. The AMOEBA + NN result is therefore within 1.31 kcal mol^−1^ discrepancy of experimental values. Free energy differences Δ*G* between windows and cumulative Δ*G* are shown in Fig. 9.

**Fig. 9:**
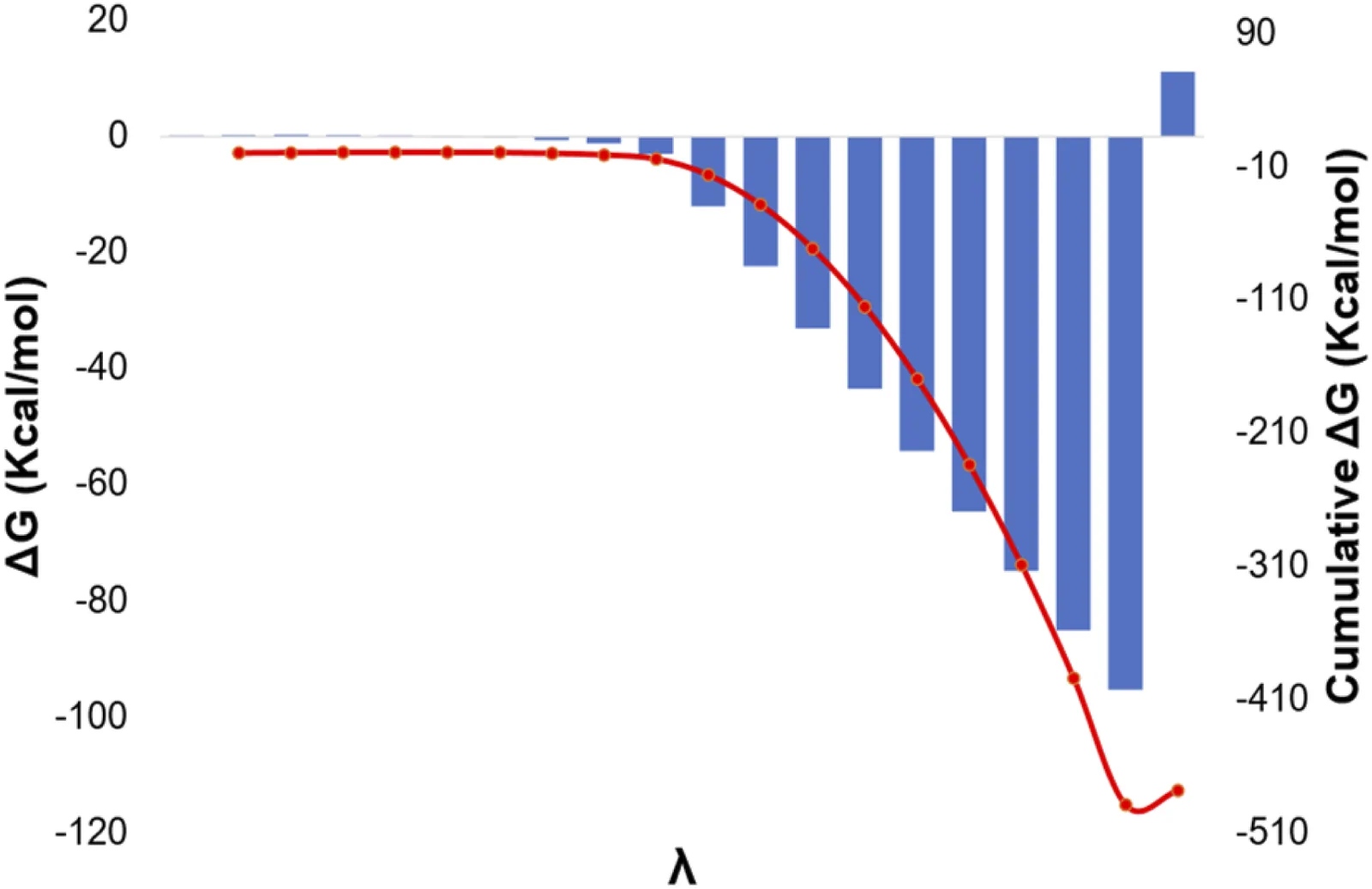
Cumulative Δ*G* and Δ*G* between windows. *λ* = 0 represents no interaction between ions and solvents and *λ* = 1 represents full interactions (vdW, electrostatic, and NN).

Taken together, these results demonstrate that the AMOEBA + NN model provides a consistent description of Cu^2+^ solvation across structural, dynamic, and thermodynamic observables. Compared to classical AMOEBA calculation, the inclusion of the NN term introduces a manageable computational increase of ∼20% (Table S1). In particular, the model captures Jahn–Teller distortion, fast ligand exchange dynamics, and accurate hydration free energies within a unified framework, highlighting its advantage over classical force fields for transition metal systems.

## 4 Conclusion

In this study, we develop and validate an integrated AMOEBA + NN potential to investigate the structural, dynamic, and thermodynamic properties of the Cu^2+^ ion in diverse solvation environments. By synergizing a physically grounded polarizable force field with a neural network correction, the model effectively overcomes the inherent limitations of classical non-polarizable and conventional polarizable descriptions for transition metal ions.

Our findings demonstrate that the AMOEBA + NN model accurately reproduces quantum-mechanical association energies and equilibrium geometries for a wide range of coordination states. Notably, the model successfully captures the electronically driven Jahn–Teller distortion in both geometry optimization and molecular dynamics simulations as evidenced by the peak splitting in radial distribution functions and the characteristic axial elongation in six-coordinate complexes. In ammonia–water solvent mixtures, it successfully retains the most stable Cu(NH_3_)_4_(H_2_O)_2_ configurations.

Dynamically, the Cu^2+^–NH_3_ system exhibits highly labile coordination, characterized by rapid ligand exchange between the first and second solvation shells with a truncated average residence time of 66.52 ps. This stands in sharp contrast to the H_2_O system, where the exchange is slower by two to three orders of magnitude, highlighting the significantly stronger binding affinity of water to Cu^2+^. Furthermore, the thermodynamic accuracy of the model was confirmed by evaluating the hydration free energy calculation, yielding a value of −477.59 ± 0.50 kcal mol^−1^, in excellent agreement with corrected experimental data.

Beyond the present study, the AMOEBA + NN framework represents a step toward a new class of hybrid potentials that explicitly compensate for short-range physics to treat local electronic complexity. The present results demonstrate that key electronic structure-driven phenomena, such as Jahn–Teller distortions and ligand exchange dynamics, can be recovered within a classical simulation framework when augmented by targeted machine learning corrections. This perspective suggests a broader paradigm for modeling transition metal systems, where neural networks act not merely as fitting tools but as physically motivated corrections to well-defined classical baselines. Such an approach holds particular promise for extending accurate simulations to systems currently beyond the reach of conventional methods, including metalloproteins, reactive solution environments, and multiscale catalytic processes. Multi-copper active sites represent an important future extension of this work, but they involve Cu–Cu coupling, bridging ligands, and possible spin or mixed-valence effects that are beyond the scope of the present mononuclear Cu^2+^ model. As the availability of high-quality quantum data continues to grow, this hybrid framework provides a natural pathway toward increasingly accurate, transferable, and computationally tractable models for complex chemical systems.

## Author contributions

P. R. and J. W. P. conceptualized the project. Z. H. generated the datasets, performed the model training and formal analysis, and wrote the original manuscript. Y. W. modified the software framework and implemented the functional modules. S. C. generated a portion of the datasets. All authors contributed to revision and edition of the draft.

## Conflicts of interest

Jean-Philip Piquemal, Jay W. Ponder, and Pengyu Ren are co-founders of Qubit Pharmaceuticals. The other authors declare no competing interests.

## Supplementary Material

SC-017-D6SC03899A-s001

SC-017-D6SC03899A-s002

SC-017-D6SC03899A-s003

SC-017-D6SC03899A-s004

SC-017-D6SC03899A-s005

SC-017-D6SC03899A-s006

SC-017-D6SC03899A-s007

SC-017-D6SC03899A-s008

## Data Availability

Simulation coordinates, NN model, training details and model performance results are available in the GitHub repository: https://github.com/prenlab/amoeba_nn. Supplementary information (SI): Table S1: computational cost of NN on an RTX 4090 graphics card. Table S2: number of samples and geometry distribution for each configuration. Fig. S1: Cu–L distance and X–Cu–X angular distribution of training set. Fig. S2: training set based on composition and generation source. Fig. S3: principal component analysis of Cu-centered AEVs for Cu(NH_3_)_*n*_ and Cu(H_2_O)_*n*_ systems. Fig. S4: scatter plots of neural network prediction *vs.* MP2 reference force components. Fig. S5: residence time distribution of ammonia/water with Cu^2+^. Fig. S6: rigid scan potential profile of a single ligand along Cu–X distance in a Cu(X)_6_^2+^ system. The *ab initio* calculation input file of single point energy calculation and molecular dynamics simulation. See DOI: https://doi.org/10.1039/d6sc03899a.
