# Supplementary material for: Incorporating a neural network into the AMOEBA polarizable force field for ligand field effects of Cu2+ ions
Source: Chem Sci. 2026 Jul 23;17(37):18001–12. doi: 10.1039/d6sc03899a (PMC13454936; doi:10.1039/d6sc03899a)

Supplementary Information (SI) for Chemical Science.
This journal is © The Royal Society of Chemistry 2026

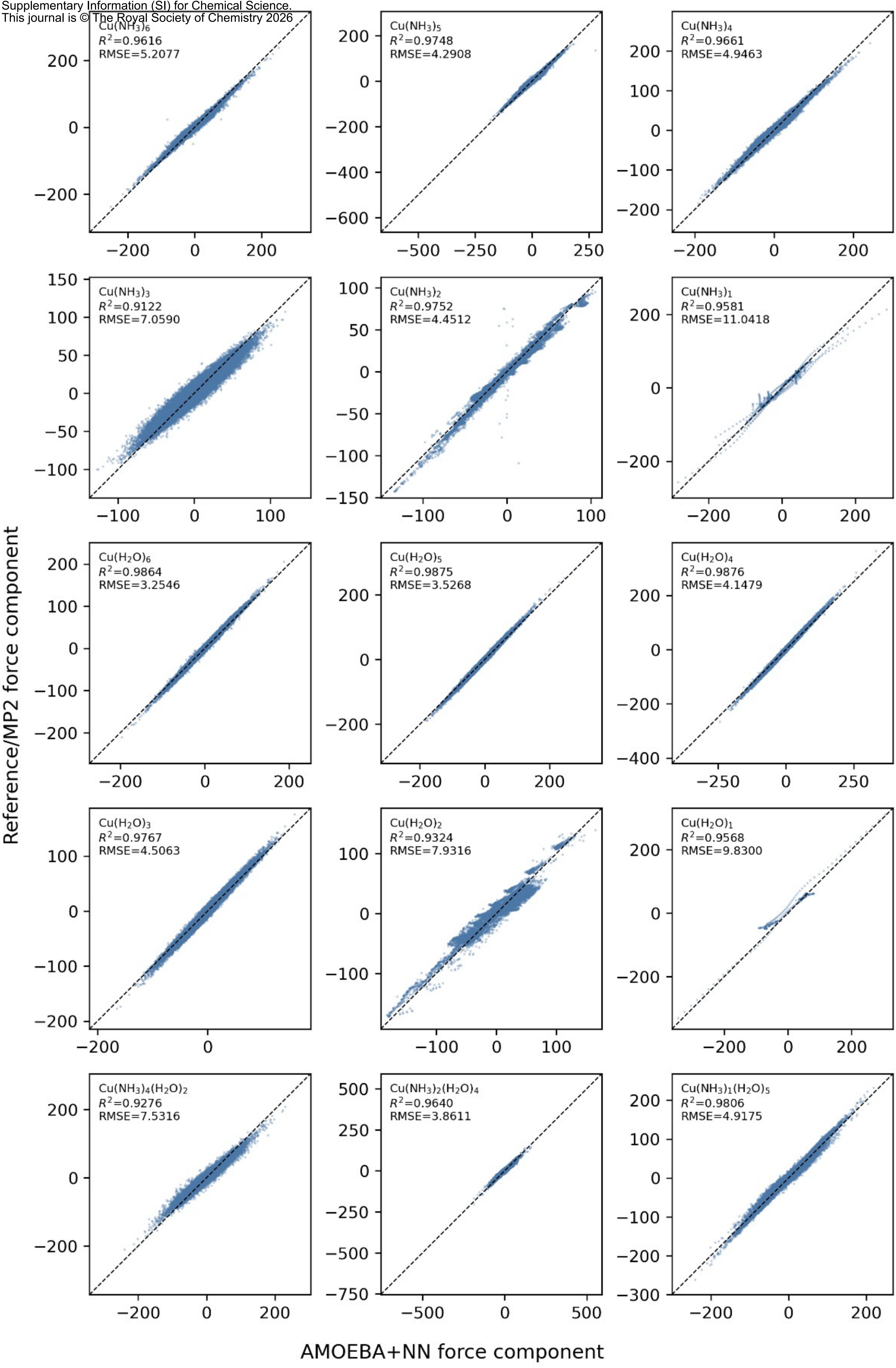

Supplement: Supplementary file 4 [file SC-017-D6SC03899A-s005.pdf]
