# Supplementary material for: Incorporating a neural network into the AMOEBA polarizable force field for ligand field effects of Cu2+ ions
Source: Chem Sci. 2026 Jul 23;17(37):18001–12. doi: 10.1039/d6sc03899a (PMC13454936; doi:10.1039/d6sc03899a)
Supplement: Supplementary file 7 [file SC-017-D6SC03899A-s008.pdf]

Supplementary Information (SI) for Chemical Science.
This journal is © The Royal Society of Chemistry 2026

Supporting Information:

Incorporating Neural Network in the AMOEBA Polarizable Force Field for Ligand Field Effects of Cu^{2+} Ion

Zhecheng He,[*a,e] Yanxing Wang,[a] , Shubham Chatterjee,[b] Jean-Philip Piquemal,[c] G. Andrés Cisneros,[b,d] Jay W. Ponder,[e] Pnegyu Ren[a]

a. Department of Biomedical Engineering, The University of Texas at Austin, Austin, Texas 78712, United States

b. Department of Chemistry and Biochemistry, The University of Texas at Dallas, Richardson, Texas 75080, United States

c. Sorbonne Université, LCT, UMR 7616 CNRS, Paris, France

d. Department of Physics, The University of Texas at Dallas, Richardson, Texas 75080, United States

e. Department of Chemistry, Washington University in St. Louis, St. Louis, Missouri 63130, United States

Table of Contents

Table S1 2
Table S2 3
Fig. S1 4
Fig. S2 5
Fig. S3 6
Fig. S4 7
Fig. S5 8
Fig. S6 9
Ab initio calculation input 10

	NH_3 (2993 atoms)	H_2O (2713 atoms)	NH_3/H_2O (2651 atoms)
AMOEBA	101.81 ns/day	120.27 ns/day	114.14 ns/day
AMOEBA+NN	85.87 ns/day	100.14 ns/day	96.64 ns/day

Table S1 Computational efficiency of neural network implementation, each system contains 1 Cu ion and bulk of solvent. Mixture contains 325 NH_3 and 450 H_2O. All simulations are conducted on RTX 4090 graphic card.

	No. configurations	Cu-L distance range (5% - 95%)	X-Cu-X Angle range (5% - 95%)
$Cu(NH_3)_6$	104332	2.05 - 2.58	82.21 - 177.03
$Cu(NH_3)_5$	103182	2.04 - 2.34	85.20 - 169.37
$Cu(NH_3)_4$	77315	1.99 - 2.20	87.62 - 162.94
$Cu(NH_3)_3$	15000	1.97 - 2.13	103.04 - 138.14
$Cu(NH_3)_2$	2310	1.75 - 2.65	94.50 - 175.50
$Cu(NH_3)_1$	151	1.58 - 2.92	n/a
$Cu(H_2O)_6$	50412	1.95 - 2.38	81.67 - 176.54
$Cu(H_2O)_5$	58630	1.94 - 2.22	84.67 - 171.07
$Cu(H_2O)_4$	60001	1.90 - 2.05	84.94 - 176.60
$Cu(H_2O)_3$	15000	1.85 - 1.99	94.31 - 157.65
$Cu(H_2O)_2$	2310	1.70 - 2.60	94.50 - 175.50
$Cu(H_2O)_1$	151	1.58 - 2.92	n/a
$Cu(NH_3)_4(H_2O)_2$	16500	2.03 - 2.65	79.56 - 174.11
$Cu(NH_3)_2(H_2O)_4$	28008	2.00 - 2.42	81.34 - 177.11
$Cu(NH_3)_1(H_2O)_5$	21000	1.95 - 2.44	80.35 - 174.79

Table S2 Number of samples and geometry distribution for each configuration.

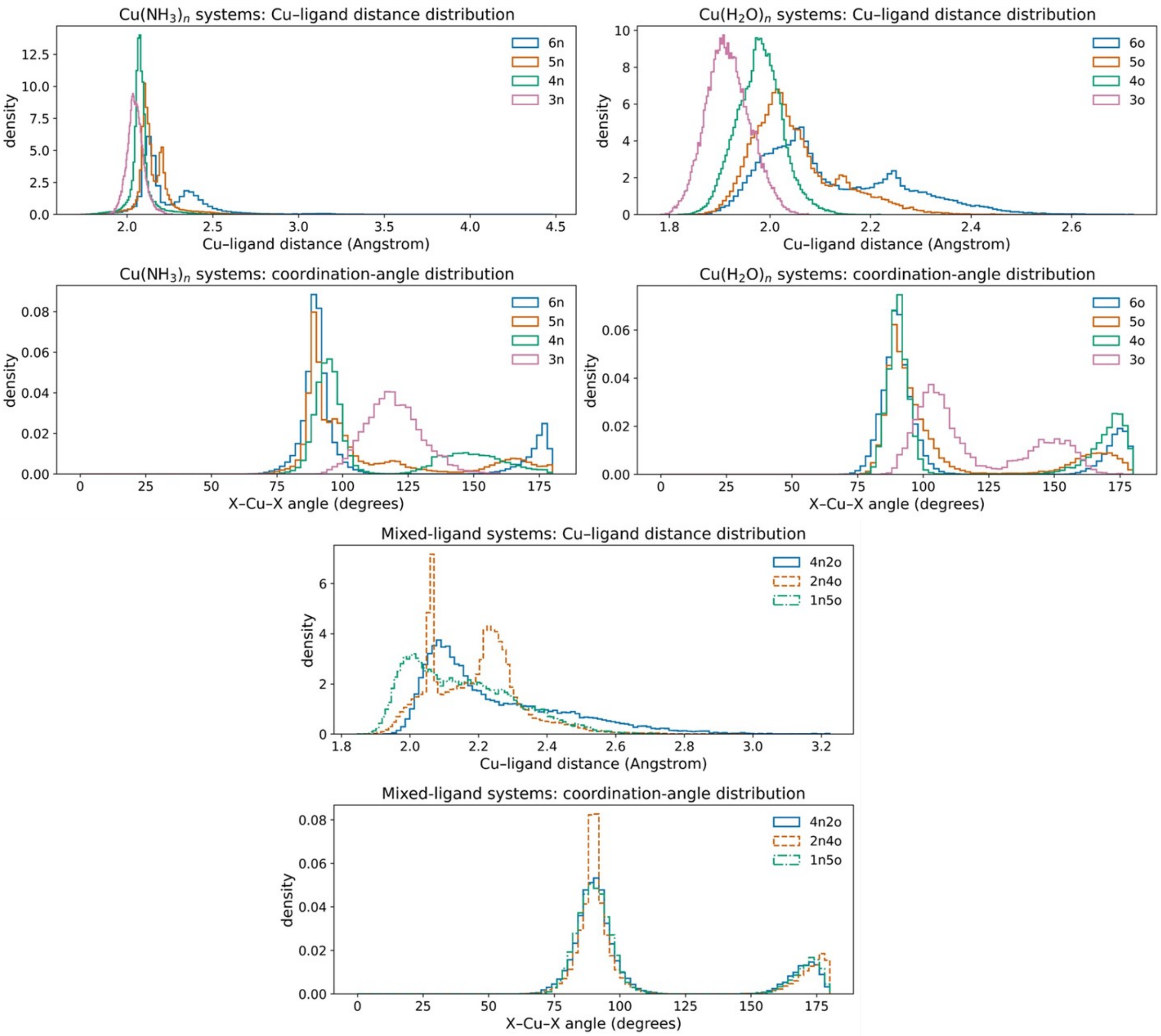

Fig. S1 Cu-L distance and X-Cu-X angular distribution of training set.

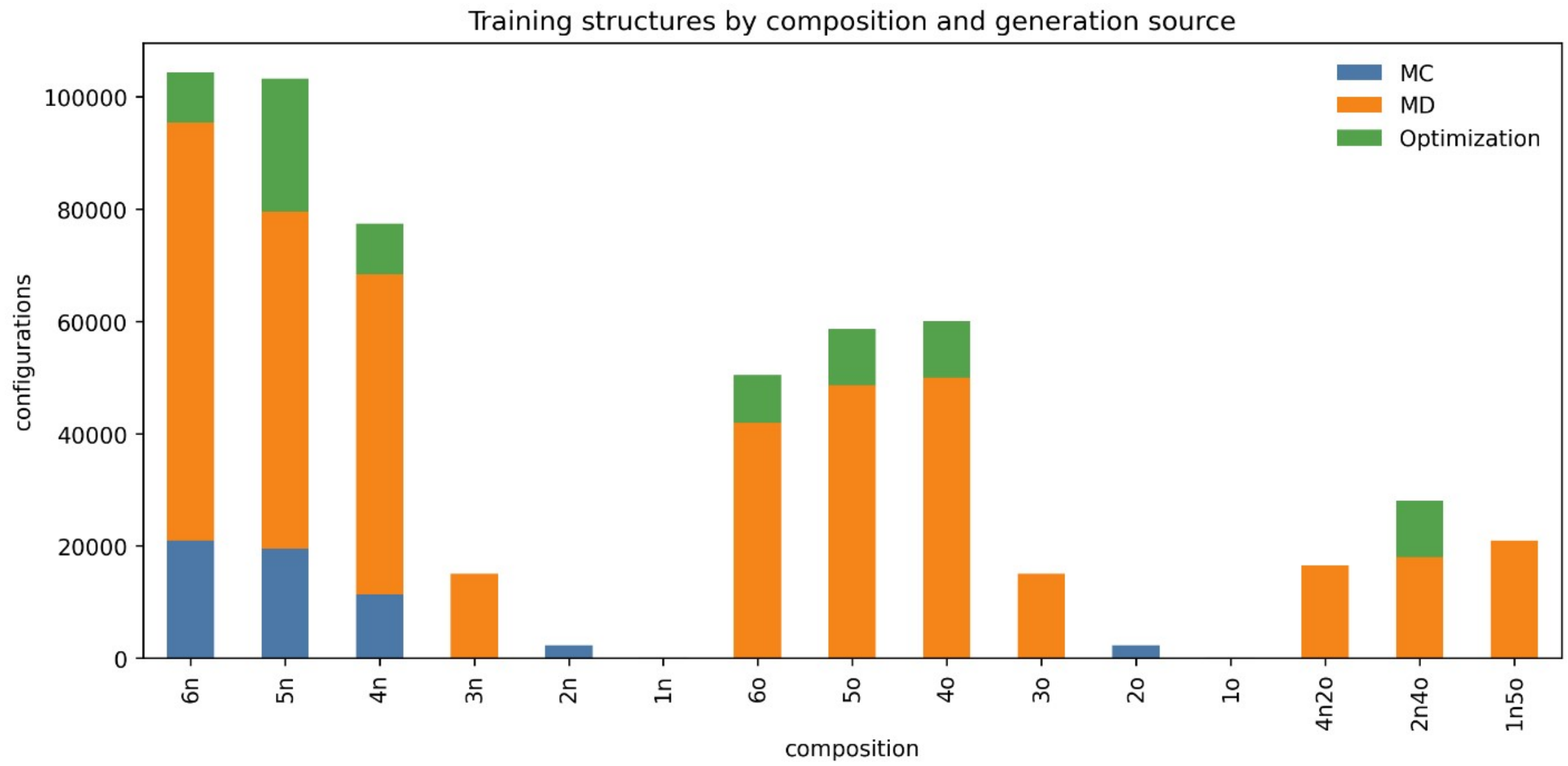

Fig. S2 Training set by composition and generation source.

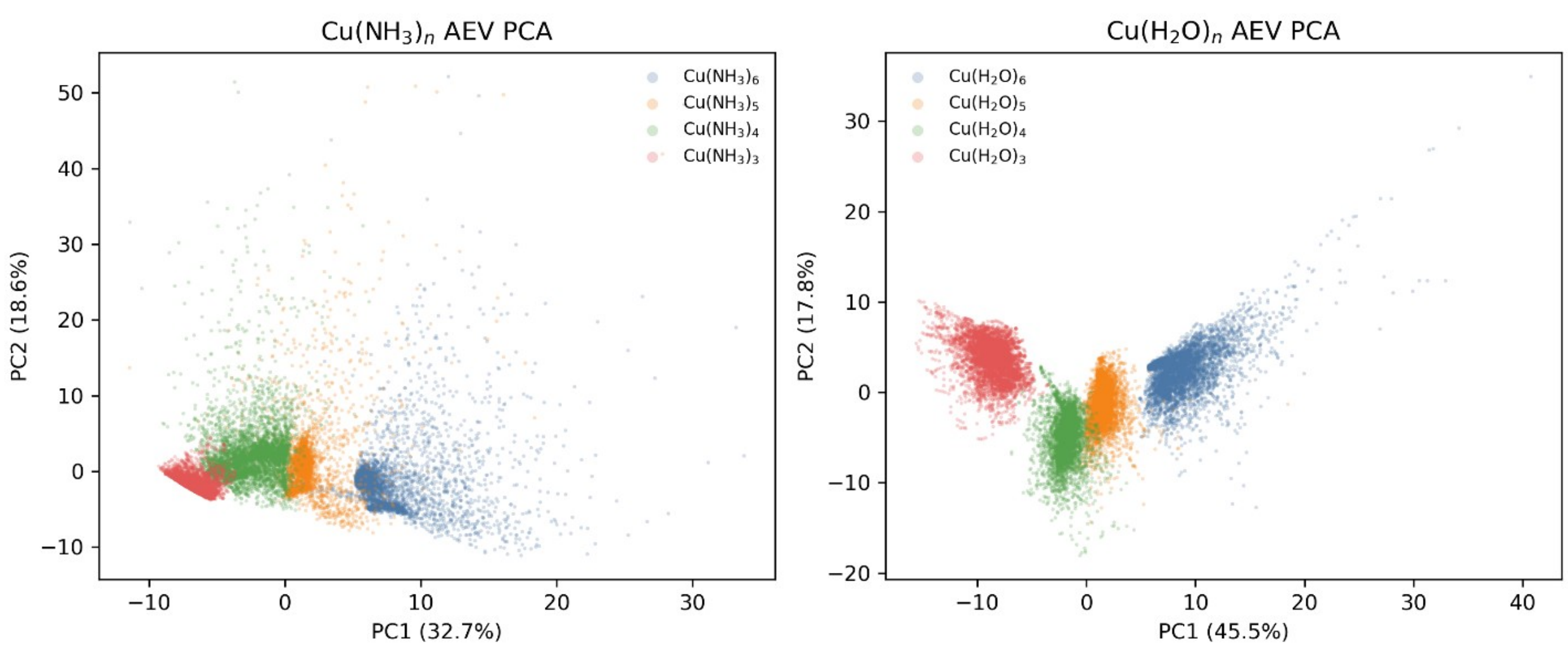

Fig. S3 Principal component analysis of Cu-centered AEV for $Cu(NH_3)_n$ and $Cu(H_2O)_n$ systems

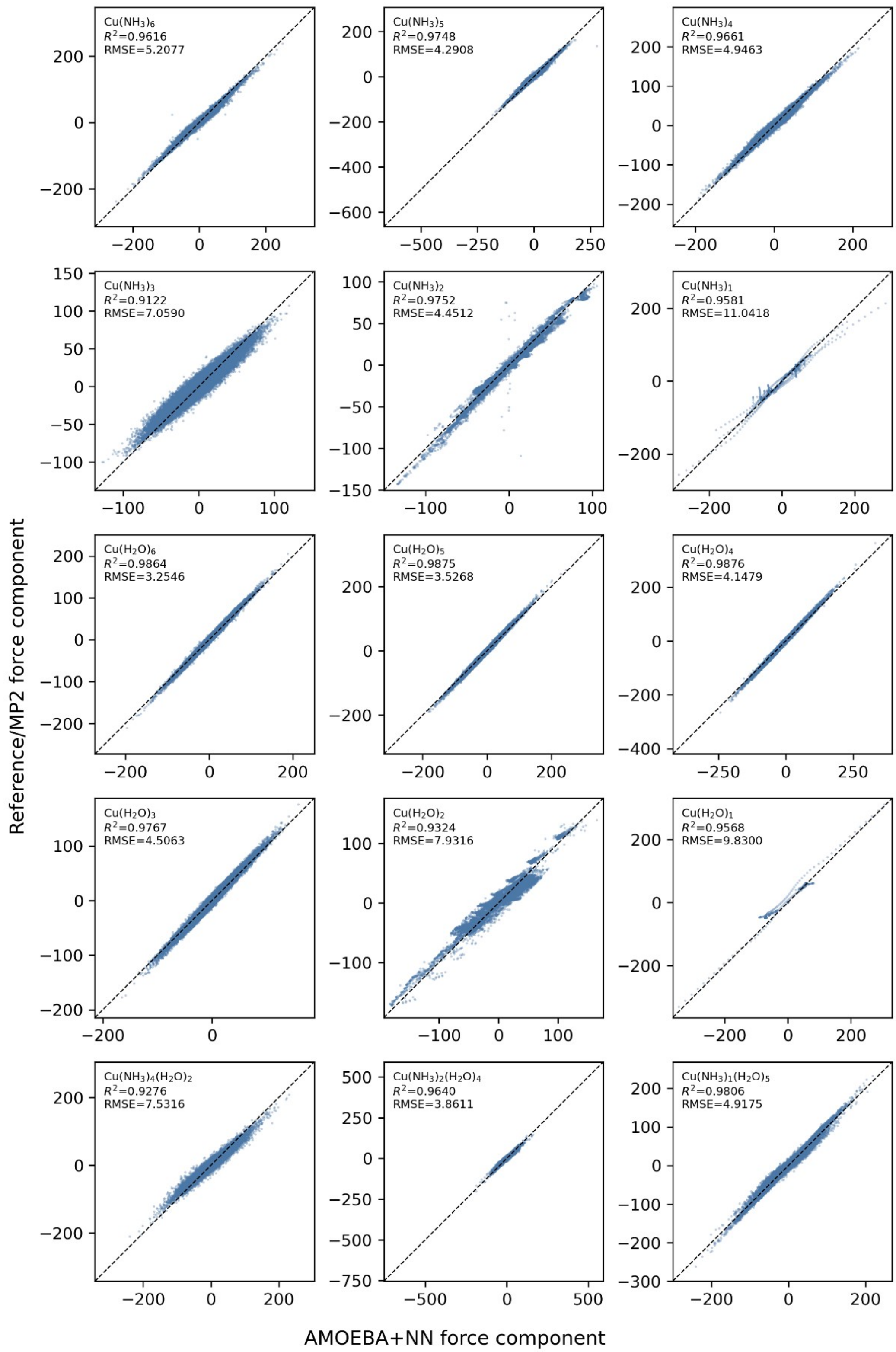

Fig. S4 . Scatter plots of neural network prediction vs MP2 reference force components. All forces components are in kcal/(mol·Ang). The dotted line denotes the identity line (y = x).

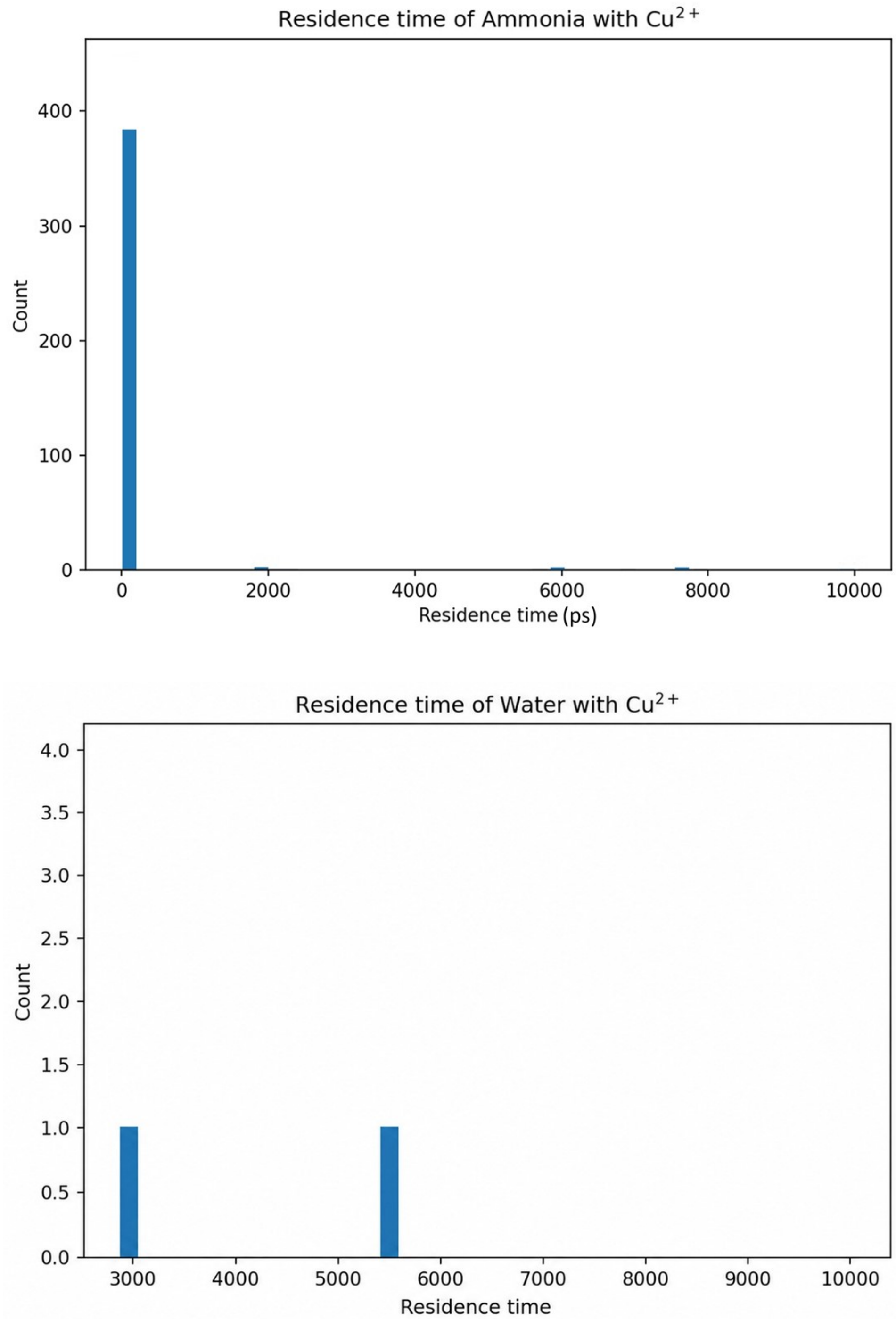

Fig. S5 Residence time distribution of ammonia/water with Cu^{2+} in AMOEBA+NN molecular dynamics simulations.

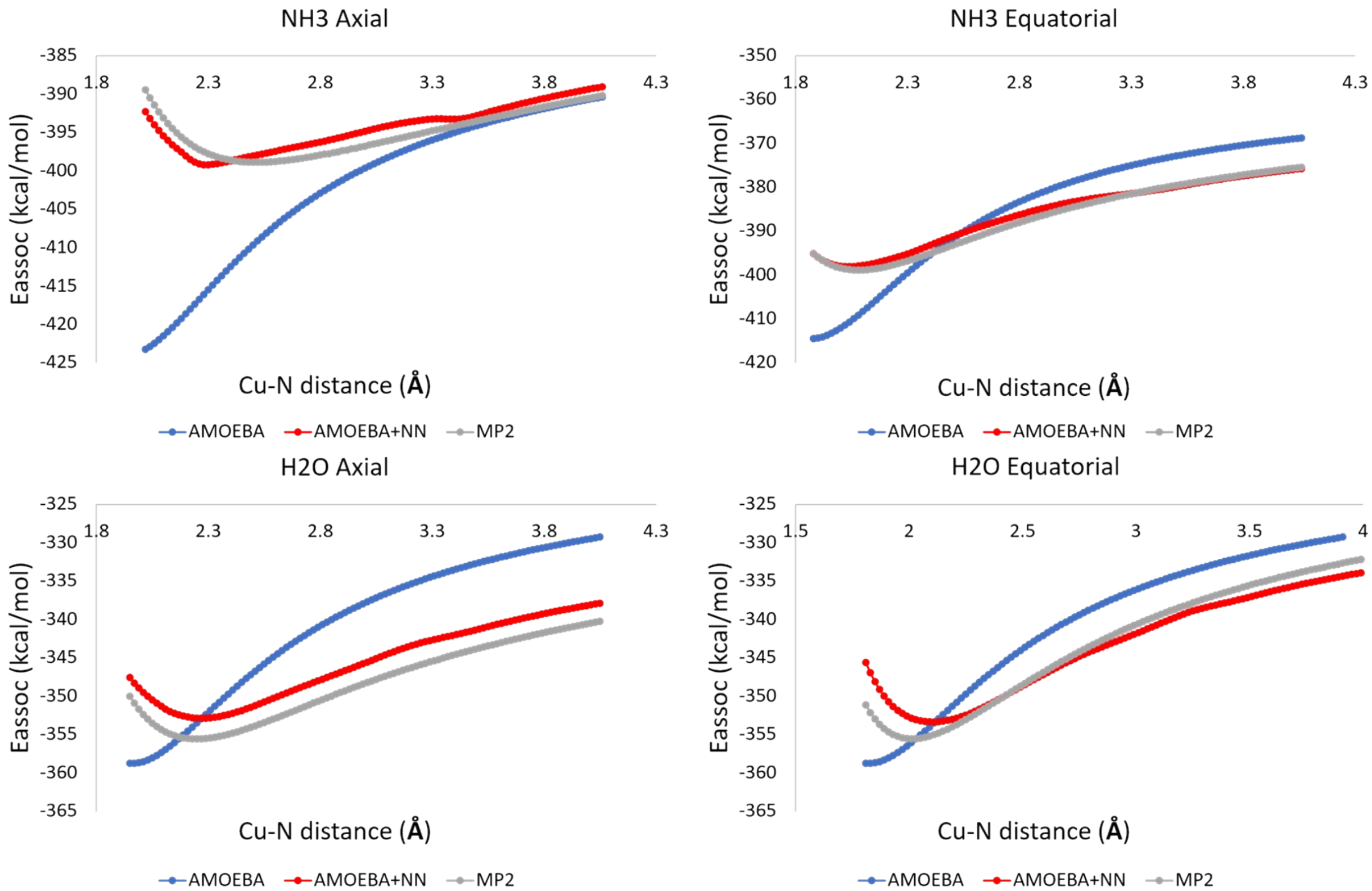

Fig. S6 Rigid scan of a single ligand along Cu-X distance in a $Cu(X)_6^{2+}$ system. X =NH_3, H_2O

Ab initio calculation input

The input parameters of the levels of theory and basis sets used in the ab initio calculations for training data invoked by ORCA-6.1.1 are as the follows:

```
!RI-MP2 DEF2-TZVP DEF2-TZVP/c DEF2/j RIJCOSX engrad
%pal nprocs 1 end
%maxcore 1000
%output
printlevel mini
print [ P_OrbEn ]  0
print [ P_SCFIterInfo ] 0
end
%scf Shift Shift 0.25 ErrOff 0.1 end end
* xyzfile 2 2 start.xyz
```

The input parameters of the levels of theory and basis sets used in the ab initio calculations for training data invoked by TeraChem are as the follows:

```
# General options
coordinates           start.xyz
charge                2
spinmult              2
run                   md
method                uwb97xd3 (or uhf)
basis                 6-31gs_ldz
scrdir                scr
# Output options
bond_order_list       yes
bond_order_thresh     0.1
# MD options
timestep              1.0
nstep                 50000
thermostat            langevin
tinit                 200 (or 298)
t0                    200 (or 298)
lnvtime               1000
seed                  31332
# SCF options
scf                   diis+a
maxit                 100
convthre              1e-4
purify                no
mixguess              0.0
# Technical options
```

```
precision            mixed
threspdp             1e-4
dftgrid              1
```
